# Effects of psychological intervention on outcomes of critically ill patients and their families: a systematic review and meta-analysis

**DOI:** 10.3389/fmed.2026.1739015

**Published:** 2026-02-09

**Authors:** Qinqin Li, Tingrui Wang, Zhangyi Wang, Jiajia Yin, Yan Liu, Zihan Zhou, Gang Lei, Zhenfa Li, Jie Yang, Zhigang Zhang, Li Yao

**Affiliations:** 1 School of Nursing, Guizhou Medical University, Guiyang, Guizhou, China; 2 Clinical Medical Technology Demonstration Base for Emergency Treatment of Chest Pain in Hunan Province, The Affiliated Hengyang Hospital of Hunan Normal University and Hengyang Central Hospital, Hengyang, China; 3 President’s Office, Hengyang Fifth People’s Hospital, Hengyang, China; 4 Department of Critical Care Medicine, The First Hospital of Lanzhou University, Lanzhou, Gansu, China; 5 School of Nursing, Lanzhou University, Lanzhou, Gansu, China; 6 Department of Respiratory and Critical Care Medicine, The Affiliated Hospital of Guizhou Medical University, Guiyang, Guizhou, China

**Keywords:** critical care nursing, family, intensive care units, patients, psychological intervention

## Abstract

**Aims:**

To evaluate the effectiveness of psychological interventions in alleviating Post-Intensive Care Syndrome (PICS) in ICU patients and PICS-Family (PICS-F) in their families.

**Design:**

Systematic review and meta-analysis of randomized controlled trials (RCTs).

**Data sources:**

PubMed, Web of Science, Cochrane Library, and Embase were searched from database inception until December 2nd, 2025.

**Review methods:**

Two reviewers independently screened the studies, extracted the data, and evaluated the risk of bias of the evidence. A systematic review and meta-analysis approach was employed, integrating both qualitative synthesis and quantitative statistical methods to analyze the included RCTs. We included RCTs that compared any form of psychological intervention against any type of control intervention.

**Results:**

A total of 25 RCTs involving 3, 849 participants were included. Among them, 22 studies included 3, 070 ICU patients, and 5 studies included 779 family members of ICU patients. The main findings are summarized as follows: (1) patients: psychological interventions demonstrated potential in reducing anxiety symptoms, with effects sustained into short-term follow-up. While depression improved immediately post-intervention, this benefit was not maintained at follow-up. No significant effects were observed for sleep quality, PTSD, or quality of life. (2) families: no statistically significant improvements were found across all assessed outcomes.

**Conclusion:**

This meta-analysis comprehensively evaluates psychological interventions for ICU patients and their families. Preliminary evidence suggests that specific interventions may improve anxiety and depression in patients, though effects varied and evidence is limited by small trials and heterogeneity. No significant effects were found for family outcomes. Current evidence remains insufficient to draw definitive conclusions, highlighting the need for larger, high-quality trials with clearly defined interventions.

**Systematic review registration:**

https://www.crd.york.ac.uk/PROSPERO/view/CRD420251003303, CRD420251003303.

## 1 Introduction

With the rapid advancement of critical care medicine on a global scale, the survival rates of critically ill patients have been progressively improving on an annual basis (1). Nevertheless, discharge from the intensive care unit (ICU) does not necessarily signify full recovery. Survivors of ICU treatment often endure a range of functional impairments encompassing psychological, physiological, cognitive, and social dimensions following their transfer, a condition collectively referred to as post-intensive care syndrome (PICS) (2). Research indicates that over 50% of critically ill patients experience varying degrees of PICS symptoms following hospital discharge, significantly impairing their quality of life and daily functioning (3). Furthermore, family members of patients admitted to the ICU experience considerable stress and burdens. These challenges encompass high treatment costs, the abrupt admission of their loved ones to the ICU due to critical illness, restricted visiting hours, unfamiliarity with the ICU environment, anxiety regarding uncertain prognoses, and the responsibility of making medical decisions on behalf of the patient (4). Such prolonged and intense stressors may result in analogous physiological, psychological, and social functional disorders in family members akin to those experienced by patients. This phenomenon is referred to as Post-ICU Syndrome-Family Members (PICS-F) (5).

To enhance the prognosis and quality of life for patients in the ICU and their families, a range of psychological interventions have been proposed and studied. These interventions include ICU diaries (6), mindfulness-based stress reduction therapy (7), cognitive behavioral therapy (CBT) (8), relaxation techniques (9), and psychological education (10). A growing body of clinical trials has investigated the impact of these intervention strategies on both the physical and psychological well-being of ICU patients and their families. Several systematic reviews and meta-analyses have summarized the available evidence, indicating potential benefits of certain interventions for specific outcomes (11, 12). Previous systematic reviews, however, have predominantly focused on psychological intervention strategies in isolation. These reviews have often limited their focus to the patient population alone, thereby overlooking the interconnected psychological experiences of both patients and their families. In fact, PICS-F may also cause patients to experience a variety of health problems, a decline in their quality of life, and even affect their ability to resume normal daily activities (13). Currently, a comprehensive review that integrates and critically evaluates existing psychological intervention measures for ICU patients and their families remains absent. As a result, the existing body of literature is fragmented, and the comparative effectiveness of various psychological intervention methods remains ambiguous.

The objective of this systematic review and meta-analysis is to comprehensively synthesize existing randomized controlled trials (RCTs) that primarily assess the efficacy of psychological interventions on anxiety, depression, post-traumatic stress disorder (PTSD), sleep, and quality of life among ICU patients and their families. This analysis aims to offer critical insights for clinical management, thereby to inform strategies for improving in the health outcomes of both patients and their families.

## 2 Methods

### 2.1 Study design

This study was registered on 3 March 2025 with the PROSPERO database of systematic reviews (CRD420251003303) and reported following the Preferred Reporting Items for Systematic Reviews and Meta-Analysis (PRISMA) guidelines (14). During the conduct of the review, amendments were made to the original protocol and formally updated in the PROSPERO registration record. The amendments were: (1) Search Strategy: Due to institutional database access limitations, the search was confined to PubMed, Web of Science, Cochrane Library, and Embase. (2) Outcome Scope: To ensure a focused and feasible synthesis, the protocol was amended to explicitly define psychological outcomes as the primary focus of this review, while the original version included both psychological and physiological outcomes. This manuscript reports the findings based on the final, amended protocol.

### 2.2 Data sources and search strategy

We searched PubMed, Web of Science, Cochrane Library and Embase from database inception to 4 March 2025 and updated our search on December 2nd, 2025. Our search strategy integrated the terms “psychological intervention,” “critical,” and “clinical trial,” employing a combination of Medical Subject Headings (MeSH) and keywords to conduct the search. We conducted a concurrent search for references pertaining to pertinent systematic reviews and clinical guidelines. Comprehensive retrieval strategies for all databases are detailed in Supplementary 1.

### 2.3 Study selection

We have selected the full-text studies based on the following criteria: (1) types of studies: we included RCT. We did not limit our studies based on the duration of follow-up. We excluded short reports, research letters, conference abstracts, and studies that had not been published in their entirety in peer-reviewed scientific journals. (2) Types of participants: we included who were adult patients (aged > 18 years) admitted to the ICU for at least 24 h, or their family members. In our study, a family member is defined as the individual within the familial unit who is most actively engaged in the treatment and care decision-making processes for the patient, irrespective of the presence of a consanguineous relationship with the patient (15). (3) Types of interventions: we included studies comparing psychological interventions (either alone or in combination with other treatments) with any comparator intervention. Psychological interventions were defined as all types of counseling, psychoeducation, social support, or therapy that are based on psychological principles and aimed at improving general well-being (16). We also included interventions that were explicitly defined as “psychological interventions” by the study authors (17). (4) outcome measures: the primary outcome measures comprised anxiety, depression, and sleep quality, while the secondary outcome measures encompassed PTSD and quality of life. For all outcome indicators of interest, we extracted the relevant result data from all available follow-up time points. We imposed no restrictions on the publication language. Articles from all databases were imported into EndNote X9 for organization. Following the removal of duplicates, an initial screening was conducted by reviewing the titles and abstracts. Subsequently, a more comprehensive screening was performed through read full-text. The process was conducted independently by two researchers. In instances of disagreement, consensus was achieved through consultation with a third researcher. Furthermore, we excluded any articles for which full texts were not accessible.

### 2.4 Data extraction

Two researchers independently employed standardized forms to extract data separately. Any disagreements were resolved through consultation with a third researcher. The extracted data encompassed: (1) fundamental information about the literature, including the first author, country, and publication year; (2) research design details, such as the type of research design, inclusion and exclusion criteria for participants, the number of participants, and follow-up duration; (3) participant demographics, including age and gender; (4) specifics of the psychological intervention, such as the key components of both the psychological and control interventions, the provider of the intervention, as well as the dosage, frequency, and duration of the intervention; and (5) outcome data such as anxiety, depression, sleep quality, PTSD and quality of life.

For all outcomes of interest, data were extracted from all available follow-up time points. Based on the approach by Ho et al. (17), the data were categorized according to the following time intervals: pre-intervention (i.e., baseline); immediately post-intervention (i.e., at the end of treatment or within < 2 months after the intervention); short-term sustainability (from ≥ 2 months to < 6 months post-intervention); medium-term sustainability (from ≥ 6 months to < 12 months post-intervention); and long-term sustainability (≥ 12 months post-intervention).

### 2.5 Risk of bias

Two reviewers independently assessed the methodological quality of individual studies using the Cochrane Risk of Bias Tool 2.0 (18). This tool comprises five domains of bias: bias arising from the randomization process, bias due to deviations from intended interventions, bias due to missing outcome data, bias in measurement of the outcome, and bias in selection of the reported result. Each domain was classified as being at “low,” “some concerns,” or “high” risk of bias. Two authors independently assessed the risk of bias, and any discrepancies in the quality assessment were resolved through consultation with a third author.

### 2.6 Statistical analysis

All statistical analyses were performed using RevMan (Review Manager) Version 5.4.1. A two-tailed *p*-value < 0.05 was considered statistically significant for overall effects. For continuous outcomes, the treatment effect was expressed as the Standardized Mean Difference (SMD) with 95% *CI*. The magnitude of the effect size, expressed as the SMD, was interpreted according to Cohen’s proposed criteria, whereby an absolute SMD value of approximately 0.2 is considered a small effect, approximately 0.5 a medium effect, and approximately 0.8 a large effect (19). Statistical heterogeneity among the included studies was assessed using the Cochran’s Q test (*χ^2^* test) and quantified using the *I*^2^ statistic. Given the anticipated clinical and methodological diversity, a random-effects model was used as the primary analytical approach. A fixed-effect model was additionally applied for comparison only in cases of negligible heterogeneity (*I*^2^ < 30%). To explore potential sources of substantial heterogeneity (*I*^2^ ≥ 50%), pre-specified subgroup analyses were conducted based on: type of psychological intervention, ICU clinical setting, assessment tool, provider expertise, mode of delivery and follow-up duration. Between-subgroup differences were tested using the standard *χ^2^* test. Sensitivity analyses were conducted to assess the robustness of the pooled results by: Sequentially removing each individual study to examine its impact on the overall effect size. All studies with a “high-risk” quality assessment were excluded for conducting the sensitivity analysis. Comparing results between studies with a low overall risk of bias and those with a high risk of bias. If a sufficient number of studies (*n* ≥ 10) were included in a meta-analysis, potential publication bias would be assessed visually using a funnel plot and statistically using Egger’s regression test.

## 3 Results

### 3.1 Search results

**Figure 1:**
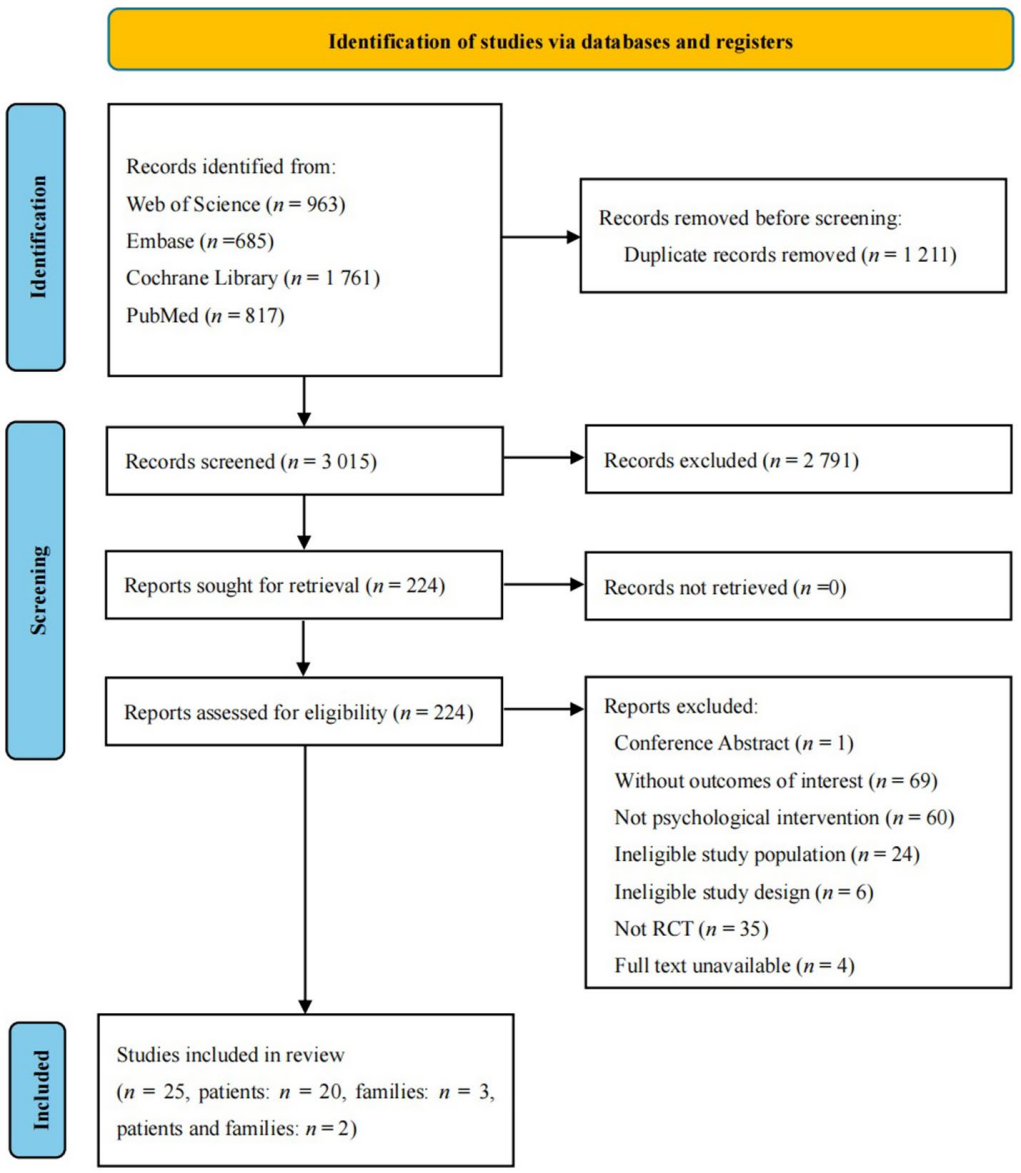
The PRISMA flow diagram.

A total of 4, 226 records were initially obtained. Following the removal of 1, 211 duplicate records and the exclusion of 2, 791 records deemed irrelevant based on a review of their titles and abstracts, 224 studies were identified as meeting the criteria for full-text screening. Of these, 199 studies failed to satisfy the inclusion eligibility standards. Consequently, 25 RCTs were ultimately included in the analysis (6–10, 20–39). Figure 1 shows the PRISMA flow diagram of study selection.

### 3.2 Characteristics of the included studies

A total of 25 studies included 3,849 participants. Among them, 22 studies included 3,070 ICU patients (6–10, 20–31, 34, 36–39), and 5 studies included 779 family members of ICU patients (6, 32, 33, 35, 37). Table 1 provides a summary of the characteristics of the studies included and Table 2 provides a detailed comparison of the characteristics of the psychological interventions.

**Table 1:** Characteristics of included studies.

Population	Author (year)	Country	Study design	Study setting	Sample size all (I/C)	Intervention	Control	Follow-up duration	Outcomes
Patients	Richardson (1997) (20)	USA	RCT	MSICU/CCU/MICU	36 (16/20)	Nursing intervention: autogenic relaxation and guided imagery	UC	NR	③
	Knowles et al. (2009) (34)	UK	RCT	ICU	36 (18/18)	ICU diaries	UC	NR	①②
	Jones et al. (2010) (36)	European countries(UK, Sweden, Italy, Denmark, Norway, Portugal)	RCT	ICUs	352 (177/175)	ICU diaries	UC	2 months	④
	Papathanassoglou et al. (2018) (22)	Republic of Cyprus	RCT	ICU	60 (30/30)	Integrative nursing intervention	UC	NR	①③
	Kredentser et al. (2018) (10)	Canada	RCT	ICU	43 (15/14/14)	ICU diary/ psychoeducation	UC	3 months	①②④
	Cox et al. (2019) (21)	USA	RCT	MSICU/CCU	62 (31/31)	Mindfulness training	Education	1,3 months	①②④⑤
	Wade et al. (2019) (23)	UK	RCT	ICUs	786 (340/446)	Nurse-Led preventive psychological intervention	UC	6 months	①②④⑤
	Garrouste-Orgeas et al. (2019) (6)	French	RCT	ICUs	339 (164/175)	ICU diaries	UC	3 months	①②④
	Lee et al. (2020) (24)	Korea	RCT	CCU	48 (24/24)	Meditation	UC	NR	③
	Nielsen et al. (2020) (37)	Denmark	RCT	MSICU	75 (36/39)	ICU diaries	UC	3 months	①②④⑤
	Rousseaux et al. (2022) (25)	Belgium	RCT	CCU	50 (25/25)	Hypnosis	UC	NR	①
	Zarghi et al. (2022) (9)	Iran	RCT	CCUs	64 (32/32)	Benson relaxation	UC	NR	①
	Wang et al. (2022) (26)	China	RCT	ICUs	106 (56/50)	VR-based intensive psychological intervention	Traditional psychological counseling	12 months	①②
	Kavakli et al. (2023) (27)	Turkey	RCT	CCU	100 (50/50)	Psychological counseling and sleep mask	UC	NR	①③
	Kutenai et al. (2023) (28)	Iran	RCT	BICU	40 (20/20)	Benson relaxation/	UC	NR	①
	Liang et al. (2023) (29)	China	RCT	SICU	152 (76/76)	Sensory stimulation intervention	UC	NR	②④
	Cox et al. (2024) (7)	USA	RCT	MSICU/CCU	247 (125/122)	Mobile mindfulness training	Face-to-face mindfulness intervention	1,3 months	①②④⑤
	Peng et al. (2024) (30)	China	RCT	ICU	80 (40/40)	Roy adaptation model nursing combined with psychological intervention	UC	NR	①②
	Gheiasi et al. (2024) (8)	Iran	RCT	CCU	90 (45/45)	Nurse-Led CBT	UC	NR	③
	Li et al. (2024) (31)	China	RCT	CCU	148 (70/78)	VR-based CBT	Standard mental health support	3 months	①③⑤
	Kim et al. (2025) (39)	Korea	RCT	SICU	96 (49/47)	Meditation	UC	NR	③
	Ozdemir et al. (2025) (38)	Turkey	RCT	CCU	60 (30/30)	Psychosocial Nursing Interventions	UC	NR	①③
Families	Jones et al. (2012) (35)	UK, Sweden	RCT	ICUs	30 (15/15)	ICU diaries	UC	2 months	④
	Cairns et al. (2019) (32)	USA	RCT	CCU	10 (5/5)	Sensation awareness focused training	UC	3 months	①②④⑤
	Garrouste-Orgeas et al. (2019) (6)	French	RCT	ICUs	563 (281/282)	ICU diaries	UC	3 months	①②④
	Nielsen et al. (2020) (37)	Denmark	RCT	MSICU	116 (56/60)	ICU diaries	UC	3 months	①②④⑤
	Petrinec (2023) (33)	USA	RCT	ICU	60 (30/30)	CBT-based mental health app	UC	2 months	①②④⑤

I/C, Intervention group/Control group; USA, the United States of America; MSICU, medical-surgical intensive care unit; CCU, Cardiac Care Unit; MICU, Medical Intensive Care Unit; SICU, Surgical Intensive Care Unit; EICU, Emergency Intensive Care Unit; BICU, Burn Intensive Care Unit; NR, not reported; UC, usual care; VR, Virtual Reality; CBT, Cognitive Behavioral Therapy.

① anxiety; ② depression; ③ sleep quality; ④ PTSD; ⑤ quality of life.

**Table 2:** Characteristics of interventions in included studies.

Population	Category	Studies	Intervention	Provider	Duration	Frequency	Delivery
Patients	Behavioral therapy (focus on behavior regulation, relaxation techniques, sensory stimulation, and non-cognitive restructuring)	Richardson (1997) (20)	Autogenic relaxation and guided imagery	Nurses	13–18 min, twice consecutively	Once daily	Face-to-face
		Zarghi et al. (2022) (9)	Benson relaxation	Trained researchers	18:00–20:00, twice consecutively	Once daily	Face-to-face
		Kutenai et al. (2023) (28)	Benson relaxation	Trained researchers	8:00–11:00, 7 consecutive days	Once daily	Face-to-face
	CBT	Gheiasi et al. (2024) (8)	CBT	Trained urses	4 consecutive weeks	Once daily	Face-to-face
		Li et al. (2024) (31)	CBT	Trained urses	7 consecutive days	Once daily	VR
	ICU diaries	Knowles et al. (2009) (34)	ICU diaries	Nurses	60 min	Once	Face-to-face
		Jones et al. (2010) (36)	ICU diaries	Nurses	–	Once	Face-to-face
		Kredentser et al. (2018) (10)	ICU diaries	Nurses	–	Once	Face-to-face
		Garrouste-Orgeas et al. (2019) (6)	ICU diaries	Nurses	–	Once	Face-to-face
		Nielsen et al. (2020) (37)	ICU diaries	Nurses	–	Once	Face-to-face
	Mindfulness and meditation	Cox et al. (2019) (21)	Mindfulness training	Psychotherapists	30 min/session	Weekly	Remote delivery
		Lee et al. (2020) (24)	Meditation	Nurses	30 min/session	On the evening of ICU admission	VR
		Rousseaux et al. (2022) (25)	Hypnosis	Hypnotist	30 min/session	Once daily, 1 day pre-op and post-op day 2	VR
		Wang et al. (2022) (26)	Meditation and relaxation	Psychotherapists	30 min/session	Twice daily (post-awakening and pre-sleep)	VR
		Cox et al. (2024) (7)	Mindfulness training	Psychotherapists	12–18 min	twice-daily	Remote delivery
		Kim et al. (2025) (39)	Meditation	Researchers	30 min, ≤7 days	Once daily	VR
	Psychoeducation	Kredentser et al. (2018) (10)	Psychoeducation	Nurses	–	Once	Face-to-face
	Psychological interventions delivered with non-psychological co-interventions	Papathanassoglou et al. (2018) (22)	Integrative nursing intervention	Trained nurses	9:30–11:30 a.m., in-hospital	Once daily	Face-to-face
		Wade et al. (2019) (23)	Nurse-Led preventive psychological intervention	Trained nurses	30 min, 3 sessions	Once daily	Face-to-face
		Kavakli et al. (2023) (27)	Psychological counseling and sleep mask	Trained Researchers	10–30 min	Once daily	Face-to-face
		Liang et al. (2023) (29)	Sensory stimulation intervention	Trained researchers	Up to 7 consecutive days	Once daily	Face-to-face
		Peng et al. (2024) (30)	Roy adaptation model nursing combined with psychological intervention	Nurses	7 consecutive days	Once daily	Face-to-face
		Ozdemir et al. (2025) (38)	Psychosocial Nursing Interventions	Nurses	45 min/sessions, started 24 h pre-op, ended post-op day 2	2 pre-op + 2 post-op sessions	Face-to-face
Families	Behavioral therapy	Cairns et al. (2019) (32)	Sensation awareness focused training	Trained researchers	15–20 min	3 consecutive days	Face-to-face
	CBT	Petrinec et al. (2023) (33)	CBT	Researchers	15 min	9 consecutive days	Remote delivery
	ICU diaries	Jones et al. (2012) (35)	ICU diaries	Healthcare staff	-	Once	Face-to-face
		Garrouste-Orgeas et al. (2019) (6)	ICU diaries	Nurses	–	Once	Face-to-face
		Nielsen et al. (2020) (37)	ICU diaries	Nurses	–	Once	Face-to-face

CBT, Cognitive Behavioral Therapy; ICU, Intensive Care Unit; VR, Virtual Reality.

Among the 22 studies on patients, 4 studies were from China (26, 29–31), 3 studies were from the United States of America(USA) (7, 20, 21), 3 studies were from Iran (8, 9, 28), 2 studies were from the United Kingdom (23, 34), 2 studies were from Korea (24, 39), 2 studies were from Turkey (27, 38), 1 study were from Belgium (25), 1 study was from Canada (10), 1 study was from the Republic of Cyprus (22), 1 study was from French (6), 1 study was from Denmark (37) and 1 study was from 6 European countries (36). The participants of 7 studies came from Cardiac Care Unit (CCU) (8, 9, 24, 25, 27, 31, 38), 7 studies came from multiple different ICUs (6, 7, 20, 21, 23, 26, 36), 1 study came from Burn Intensive Care Unit (BICU) (28), 1 study came from Surgical Intensive Care Unit(SICU) (29, 39), 1 study came from MSICU (37), and 6 studies did not specify from which ICU they came (10, 22, 30, 34). Psychological interventions vary significantly in their therapeutic components, including behavioral therapy (9, 20, 28), CBT (8, 31), ICU diaries (6, 10, 34, 36, 37), mindfulness and meditation (7, 21, 24–26, 39), psychoeducation (10), and psychological interventions delivered with non-psychological co-interventions (22, 23, 27, 29, 30, 38). The modes of delivery included face-to-face sessions (6, 8–10, 20, 22, 23, 27–30, 34, 36–38), virtual reality (VR) (24–26, 31, 39), and remote delivery (via telephone or apps) (7, 21). The duration and frequency of the psychological interventions also differed considerably.

Among the five studies conducted on the family members of ICU patients, 2 studies were from USA (32, 33), 1 study was from French (6), 1 study was from Denmark (37) and 1 study was from 2 European countries (35). The participants of 2 studies came from multiple different ICUs (6, 35), 1 study came from CCU (32), 1 study came from MSICU (37), and 1 study did not specify from which ICU they came (33). The psychological intervention related to family members can be classified into three categories based on the intervention mechanism: including behavioral therapy (32), CBT (33) and ICU diaries (6, 35, 37).

### 3.3 Quality assessment

Eighteen studies were classified as low-risk, while seven were classified as high-risk; the assessment results of the two authors were highly consistent. (1) Bias in the randomization process: 3 studies were rated as high risk (20, 25, 30). Among them, one study (20) did not clearly specify whether allocation concealment was implemented before recruitment, one study (25) provided insufficient details regarding the randomization process, and another study (30) lacked a detailed description of the randomization method and allocation concealment mechanism. (2) Bias in deviations from intended interventions: 1 study (37) was classified as a high-risk study due to the presence of cross-contamination between groups. (3) Bias in missing outcome data: 1 study (33) was rated as high-risk because it did not specify whether the missing data had been appropriately handled. (4) Bias in measurement of the outcome: the risk of bias was judged as low for all included studies. Although the primary outcomes were assessed using patient-reported measures, which are inherently subjective, all studies employed well-validated instruments (e.g., hospital anxiety and depression scale (HADS) for anxiety/depression, impact of events scale-revised (IES-R) for PTSD). The use of these standardized tools, with established reliability and validity, minimized the potential for measurement bias. (5) Bias in the selection of the reported result: all reported results were predefined in the methods section, and most of them have provided published research protocols; therefore, all 25 studies were identified as low risk. (6) other bias: 2 studies (32, 35) were rated as high risk due to an excessively small sample size.

### 3.4 Meta-analysis results

#### 3.4.1 Anxiety

##### 3.4.1.1 Patients

A total of 16 studies reported on patients’ anxiety (6, 7, 9, 10, 21–23, 25–28, 30, 31, 34, 37, 38). Among them, data from four studies (25, 26, 37, 38) could not be converted and were therefore included in the descriptive analysis. One study (27) only described baseline anxiety levels and did not report changes in anxiety after the intervention; therefore, it was excluded from the meta-analysis. The remaining 11 studies (6, 7, 9, 10, 21–23, 28, 30, 31, 34), involving 1,905 patients, were included in the quantitative synthesis.

**Figure 2:**
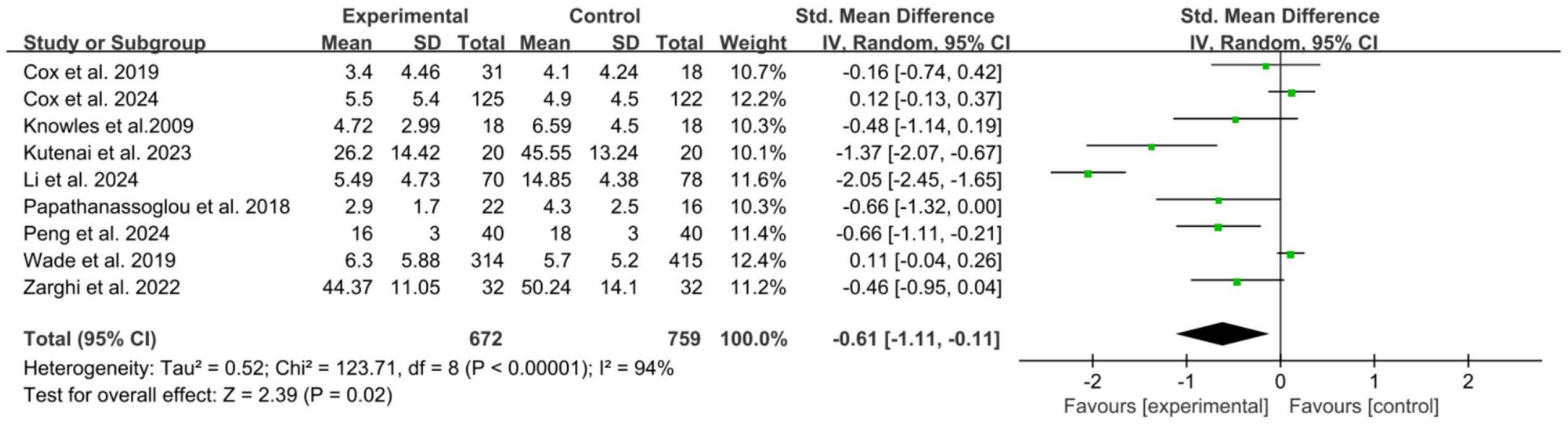
Forest plot of the meta-analysis on patients’ anxiety (post-intervention).

A total of 9 studies (7, 9, 21–23, 28, 30, 31, 34) reported the immediate effects. The meta-analysis revealed a statistically significant overall effect favoring psychological interventions in reducing anxiety symptoms compared to control conditions, with substantial heterogeneity (*I*^2^ = 94%) (Figure 2). Sensitivity analysis identified the study by Li et al. (31) as a major contributor to heterogeneity. Although its removal reduced the *I*^2^ statistic, substantial heterogeneity persisted (*I*^2^ = 80%) (Supplementary 2). To explore potential sources of heterogeneity, we conducted subgroup analyses. After sensitivity analyses were performed to reduce within-group heterogeneity, subgroup analyses by follow-up duration, ICU setting, mode of delivery, type of psychological intervention, assessment tool, and provider expertise all showed statistically significant differences between subgroups, indicating that these factors may significantly influence the effects of psychological interventions on anxiety (Supplementary 3–8).

**Figure 3:**
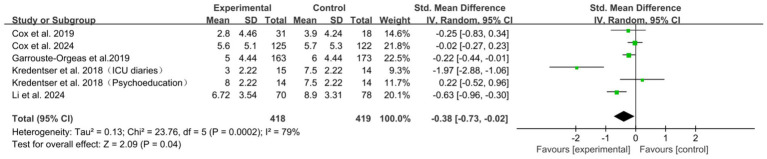
Forest plot of the meta-analysis on patients’ anxiety (short-term follow-up).

A total of 5 studies (6, 7, 10, 21, 31) assessed the sustainability of intervention effects at short-term follow-up. The meta-analysis revealed a statistically significant overall effect favoring psychological interventions in reducing short-term anxiety symptoms compared to control conditions (SMD = −0.38, 95% CI: [−0.73, −0.02], *p* = 0.04), with substantial heterogeneity (*I*^2^ = 79%) (Figure 3). Sensitivity analysis identified the study by Kredentser et al. (ICU diaries) (10) as a major contributor to heterogeneity. Its removal reduced both the overall effect size and the *I*^2^ statistic, and the statistical significance was attenuated (SMD = −0.23, 95% CI: [−0.47, 0.02], *p* = 0.07; *I*^2^ = 59%) (Supplementary 9). Subgroup analyses by assessment tool, type of psychological intervention, and ICU setting showed statistically significant differences between subgroups, suggesting these factors may explain part of the variance in intervention effects (Supplementary 10–12). Analyses by intervention provider did not show statistically significant subgroup differences (Supplementary 13).

##### 3.4.1.2 Families

Four studies (6, 32, 33, 37), involving 749 family members of ICU patients, evaluated the impact of psychological interventions on family anxiety. Among them, data from 1 study (37) could not be converted and was therefore included in the descriptive analysis. Two studies (32, 33) assessed the immediate effects. Meta-analysis showed no statistically significant difference between intervention and control groups (SMD = 0.62, 95% CI: [−1.14, 2.38], *p* = 0.49). However, substantial heterogeneity was observed between studies (*I*^2^ = 79%) (Supplementary 14). Two studies (6, 33) evaluated anxiety at short-term follow-up. Fixed-effect meta-analysis revealed no statistically significant effect of psychological interventions in reducing family anxiety symptoms (SMD = −0.02, 95% CI: [−0.18, 0.14], *p* = 0.79, *I*^2^ = 0%) (Supplementary 15).

#### 3.4.2 Depression

##### 3.4.2.1 Patients

A total of 10 studies reported on patients’ depression (6, 7, 10, 21, 23, 26, 29, 30, 34, 37). Among these, data from 2 studies (26, 37) could not be converted and were therefore included in the descriptive analysis. The remaining 8 studies (6, 7, 10, 21, 23, 29, 30, 34), involving 1,745 patients, were included in the quantitative synthesis.

**Figure 4:**
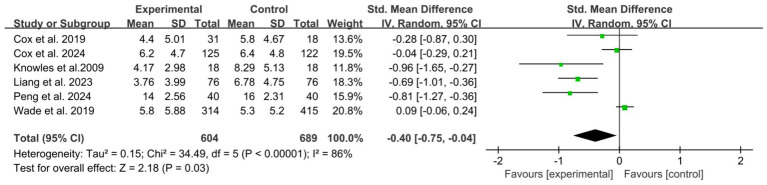
Forest plot of the meta-analysis on patients’ depression (post-intervention).

A total of 6 studies (7, 21, 23, 29, 30, 34) reported the immediate effects. The meta-analysis showed a statistically significant overall effect favoring psychological interventions compared to control conditions, with high heterogeneity (*I*^2^ = 86%) (Figure 4). After sensitivity analyses were performed to reduce within-group heterogeneity, subgroup analyses based on follow-up duration, type of psychological intervention, mode of delivery, ICU setting, assessment tool, and provider expertise. All subgroup analyses revealed statistically significant between-subgroup differences, suggesting that these factors may substantially influence intervention effects on depressive symptoms (Supplementary 16–21).

**Figure 5:**
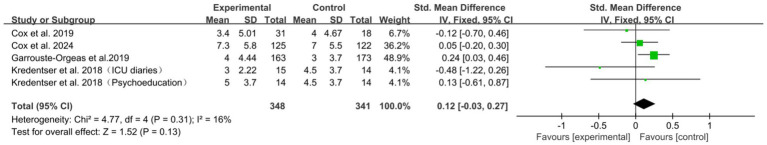
Forest plot of the meta-analysis on patients’ depression (short-term follow-up).

A total of 4 studies (6, 7, 10, 21) assessed the sustainability of intervention effects at short-term follow-up. The meta-analysis showed a non-statistically significant overall effect for the intervention compared to control conditions in improving depressive symptoms at short-term follow-up (SMD = 0.21, 95% CI: [−0.06, 0.48], *p* = 0.13), with low heterogeneity (*I*^2^ = 16%) (Figure 5).

##### 3.4.2.2 Families

Four studies (6, 32, 33, 37) involving 749 family members of ICU patients, evaluated the impact of psychological interventions on family depression. Among them, data from 1 study (37) could not be converted and was therefore included in the descriptive analysis. Two studies (32, 33) assessed the immediate effects. Meta-analysis showed no statistically significant difference between intervention and control groups (SMD = 0.53, 95% CI: [−1.00, 2.07], *p* = 0.50). Substantial heterogeneity was observed between these studies (*I*^2^ = 75%) (Supplementary 22). Two studies (6, 33) evaluated depression at short-term follow-up. Fixed-effect meta-analysis revealed no statistically significant effect of psychological interventions in reducing family depression symptoms (SMD = −0.03, 95% CI: [−0.18, 0.13], *p* = 0.72, *I*^2^ = 19%) (Supplementary 23).

#### 3.4.3 Sleep

**Figure 6:**
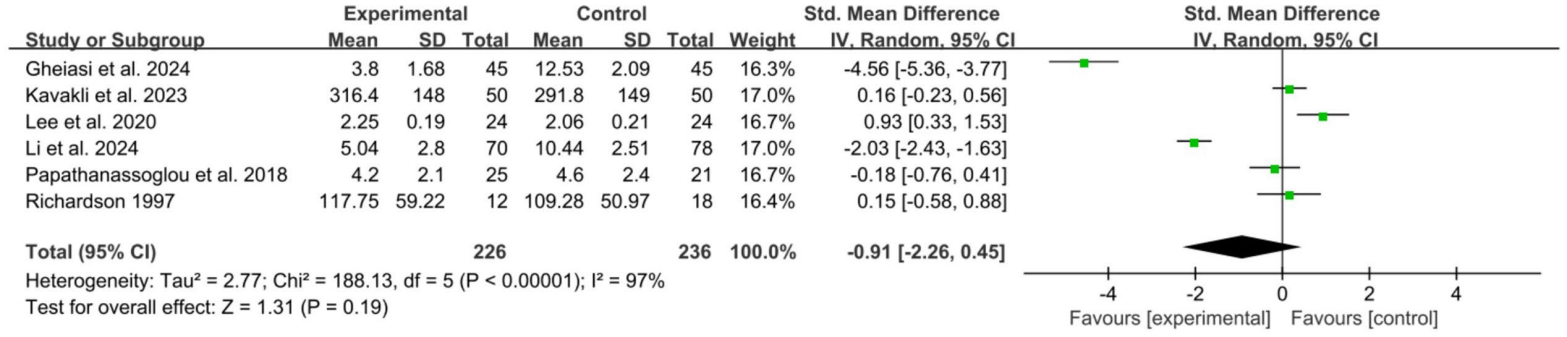
Forest plot of the meta-analysis on patients’ sleep quality (post-intervention).

A total of 8 studies (8, 20, 22, 24, 27, 31, 38, 39) reported the immediate impact on patients’ sleep quality. Among these, data from 2 studies (38, 39) could not be converted and were therefore included in the descriptive analysis. Pooled data from 6 studies (8, 20, 22, 24, 27, 31) (*n* = 462 participants) showed a large, but not statistically significant, improvement in patient’s sleep quality favoring the intervention groups, with substantial heterogeneity (*I*^2^ = 97%) (Figure 6). To explore the sources of heterogeneity, we conducted a sensitivity analysis, which revealed that after excluding the studies by Ghelasi et al. (8) and Li et al. (31), heterogeneity significantly decreased to 59%, yet the pooled effect size remained non-significant (SMD = 0.26, *p* = 0.24) (Supplementary 24). Subgroup analyses were performed based on follow-up duration, type of psychological intervention, mode of delivery, ICU setting, assessment tool, and provider expertise (Supplementary 25–30). The results indicated that only the “intervention type” subgroup analysis showed a statistically significant difference between subgroups. Within this subgroup: CBT (included 2 studies) showed a significant pooled improvement in sleep. However, substantial heterogeneity was observed within this CBT subgroup (*I*^2^ = 97%). The mindfulness and meditation subgroup (one study) also demonstrated a significant benefit (SMD = 0.93, 95% CI: 0.33 to 1.53, *p* = 0.002). The psychoeducation and behavioral therapy subgroups showed no significant effects. Other subgroup analyses did not significantly explain the sources of heterogeneity (all *P* for subgroup difference > 0.05).

#### 3.4.4 PTSD

##### 3.4.4.1 Patients

**Figure 7:**
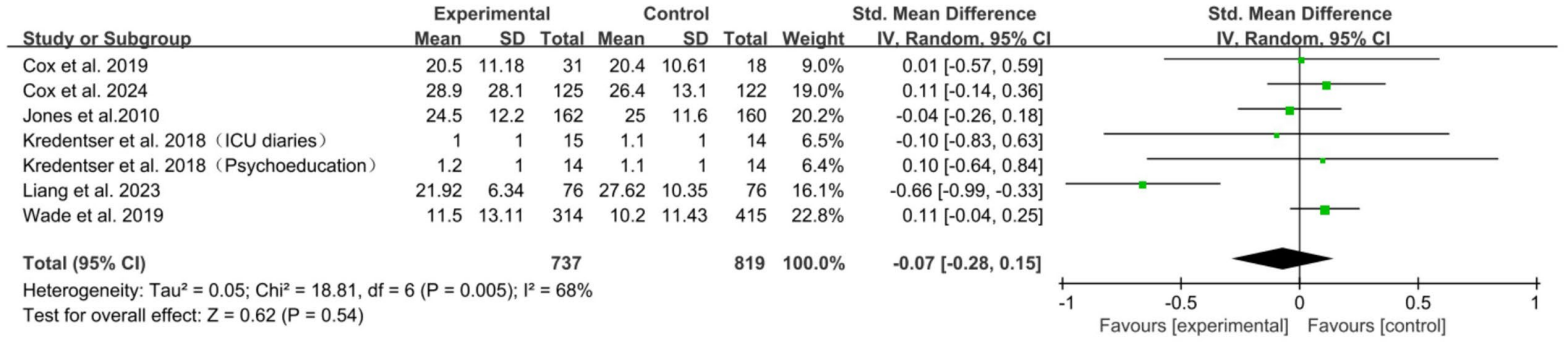
Forest plot of the meta-analysis on patients’ PTSD (post-intervention).

A total of 6 studies (7, 10, 21, 23, 29, 36) reported the immediate impact on patients’ PTSD. The meta-analysis found no statistically significant overall effect of psychological interventions on PTSD symptoms in ICU patients immediately post-intervention (SMD = −0.07; 95% CI: [−0.28, 0.15]; *p* = 0.54). Considerable heterogeneity was observed (*I*^2^ = 68%) (Figure 7). Sensitivity analysis identified the study by Liang et al. (29) as a major contributor to heterogeneity. After removing it, the heterogeneity was completely eliminated (*I*^2^ = 0%), and the pooled effect size changed slightly but remained statistically non-significant (SMD = 0.07, 95% CI: −0.04 to 0.17, *p* = 0.22) (Supplementary 31). Subgroup analyses were conducted based on follow-up duration, type of psychological intervention, mode of delivery, ICU setting, assessment tool, and provider expertise. The results indicated statistically significant between-subgroup differences in assessment tool, follow-up duration, and ICU setting, with heterogeneity primarily stemming from the study (29) using the “17-item PTSD Checklist,” those with unreported follow-up time, and those conducted in SICU settings (Supplementary 32–34). No significant differences were observed in the remaining subgroups (Supplementary 35–37).

A total of 6 studies (6, 7, 10, 21, 36, 37) assessed the sustainability of intervention effects at short-term follow-up. The meta-analysis showed no statistically significant difference between the experimental and control groups in PTSD symptom improvement (SMD = −0.04, 95% CI: −0.16 to 0.08, *p* = 0.56), with low heterogeneity among studies (*I*^2^ = 26%) (Supplementary 38).

##### 3.4.4.2 Families

Five studies (6, 32, 33, 35, 37), involving 779 family members of ICU patients, evaluated the impact of psychological interventions on family PTSD. Three studies (32, 33, 35) assessed the immediate effects. Meta-analysis showed no statistically significant difference between intervention and control groups (SMD = 0.35, 95% CI: [−0.46, 1.17], *p* = 0.40). Substantial heterogeneity was observed between these studies (*I*^2^ = 65%) (Supplementary 39). Sensitivity analysis after removing Cairns et al. (32), the heterogeneity was completely eliminated (*I*^2^ = 0%), and the pooled effect remained non-significant (*p* = 0.89) (Supplementary 40). Four studies (6, 33, 35, 37) evaluated PTSD at short-term follow-up. Meta-analysis revealed no statistically significant effect (SMD = −0.22, 95% CI: [−0.54, 0.10], *p* = 0.17). Heterogeneity was moderate (*I*^2^ = 58%) (Supplementary 41). Sensitivity analysis after removing Nielsen et al. (37), the heterogeneity decreased to 19% (*I*^2^ = 19%), and the pooled effect remained non-significant (*p* = 0.60) (Supplementary 42).

#### 3.4.5 Quality of life

##### 3.4.5.1 Patients

**Figure 8:**
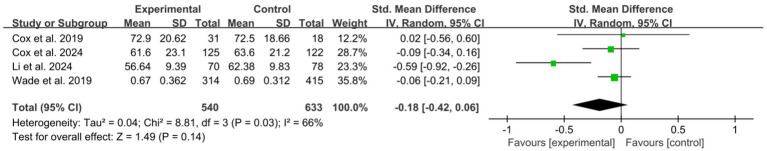
Forest plot of the meta-analysis on patients’ quality of life (post-intervention).

A total of 5 studies (7, 21, 23, 31, 37) reported the immediate impact on patients’ quality of life. Among these, data from 1 study (37) could not be converted and was therefore included in the descriptive analysis. The meta-analysis of 4 studies (7, 21, 23, 31) showed non-significant improvement in quality of life favoring the intervention groups (SMD = −0.18; 95% CI: [−0.42, 0.06]; *p* = 0.14). Considerable heterogeneity was present (*I*^2^ = 66%; *p* = 0.03) (Figure 8). Sensitivity analysis after removing Li et al. (31), the heterogeneity was completely eliminated (*I*^2^ = 0%), and the pooled effect remained non-significant (*p* = 0.31) (Supplementary 43).

At short-term follow-up (3 studies (7, 21, 31), *n* = 422 participants), there was no significant effect on quality of life (SMD = −0.06; 95% CI: [−0.25, 0.14]; *p* = 0.57). The results were perfectly consistent across studies (*I*^2^ = 0%) (Supplementary 44).

##### 3.4.5.2 Families

Three studies (32, 33, 37) evaluated the impact of psychological interventions on family quality of life. 1 study (37) could not be converted and was therefore included in the descriptive analysis. For the immediate post-intervention time, 1 study (32) provided usable data, reporting no significant difference between groups. For the short-term follow-up, 1 study (33) provided data, also indicating no significant difference between groups. Due to the insufficient number of studies with extractable data at each time point, a quantitative synthesis was not feasible.

### 3.5 Qualitative analysis

In addition to quantitative findings, qualitative insights from several studies are noteworthy. Rousseaux et al. (25) found that, compared with the virtual reality hypnosis (VRH) group, the anxiety levels of patients in the simple hypnosis group were significantly higher (*p* = 0.007). However, no significant differences in anxiety levels were observed between the other intervention groups (virtual reality group, VRH group) and the control group. Data from Wang et al. (26) showed that acute respiratory distress syndrome (ARDS) survivors receiving the Virtual Reality-Integrated Psychological Intervention (VR-IPI) exhibited consistently superior improvement trends in anxiety, depression, and PTSD symptoms at all follow-ups (3, 6, 9, 12 months) compared to those receiving Traditional Psychological Counseling (TPC). Nielsen et al. ‘s (37) study showed that diaries authored by relatives significantly reduced the relatives’ own symptoms of PTSD, but had no significant effect on the patients’ PTSD symptoms, or on the anxiety, depression, or quality of life of either patients or relatives. Ozdemir and Yilmaz’s (38) study demonstrated that structured psychosocial nursing interventions, combining cognitive behavioral therapy with sensory modulation (e.g., eye masks, earplugs), significantly reduce anxiety levels and improve postoperative sleep quality in patients undergoing open heart surgery.

### 3.6 Publication bias

Due to the number of included studies for each outcome being less than ten, the statistical power of Egger’s regression test was insufficient. Therefore, formal statistical testing was not performed, and the assessment relied primarily on visual inspection of funnel plot symmetry. The funnel plot showing the immediate effects of psychological intervention on patients’ anxiety, depression, sleep quality, PTSD, and quality of life is presented in Supplementary 45–49. (1) Depression: the funnel plot exhibited asymmetry, with a gap in the bottom-right quadrant. This suggests the presence of publication bias, indicating that small-scale studies with negative results may have been omitted, potentially leading to an overestimation of the intervention effect. (2) Sleep quality: significant asymmetry and a bottom gap were observed, indicating substantial publication bias and/or heterogeneity, which may undermine result stability. (3) PTSD: this funnel plot shows asymmetry, with only 2 studies on the left side. This indicates publication bias, meaning that studies with positive results may have been omitted, which could lead to an underestimation of the estimated intervention effect. (4) The funnel plots for anxiety and quality of life showed relatively good symmetry, indicating a lower risk of publication bias for these outcomes.

### 3.7 Sensitivity analysis

Sensitivity analysis was performed to determine the impact of each study on the pooled SMDs. The overall effect sizes did not change significantly when one study was excluded at a time, which indicated the strong stability of the results.

## 4 Discussion

### 4.1 Summary of findings

Our study evaluated the efficacy of psychological interventions on psychological distress, sleep and quality of life among ICU patients and their family members. A total of 25 RCTs involving 3, 849 participants were included. The main findings are summarized as follows: (1) For ICU patients, psychological interventions show potential in alleviating anxiety symptoms, an effect sustained from the immediate post-intervention period through short-term follow-up. For depression, a significant reduction was observed immediately post-intervention, but this improvement was not sustained at short-term follow-up. However, no statistically significant improvements were observed for sleep quality, PTSD, or quality of life at either the immediate or short-term follow-up assessments. (2) For family members of ICU patients, psychological interventions did not show statistically significant effects on anxiety, depression, PTSD, or quality of life, either immediately post-intervention or at short-term follow-up.

### 4.2 Effect of psychological interventions on patients’ anxiety

This study indicates that psychological interventions in the ICU have the potential to alleviate patients’ immediate anxiety and reduce anxiety levels in the short term. This is consistent with several previous studies, supporting the positive role of psychological intervention in the ICU (40, 41). However, the meta-analyses showed very high statistical heterogeneity, greatly limiting the reliability of applying the “overall effect” to clinical practice. We performed several subgroup analyses, revealing that follow-up duration, ICU environment, mode of delivery, type of psychological intervention, assessment tool, and provider’s expertise could significantly influence the effect size. For instance, CBT had the strongest impact, while mindfulness/meditation was not statistically significant. Face-to-face and VR interventions were effective, unlike remote interventions like telemedicine. Psychological interventions conducted in a homogeneous critical care setting (e.g., exclusively in CCUs or BICUs) were associated with a significantly greater reduction in patient anxiety compared to those implemented in mixed ICU environments. This finding may be explained by the more defined patient profiles and reduced clinical variability in homogeneous units, facilitating tailored and effective intervention delivery. It is important to underscore that these subgroup analyses are primarily exploratory and are largely derived from a limited number of studies.

Notwithstanding the heterogeneity and limitation inherent in the available evidence, the findings of this review nevertheless offer important implications for planning psychological services within the ICU. Firstly, regarding intervention selection and feasibility, the analysis suggests that even brief, manualized interventions led by nurses (such as ICU diaries or psychoeducation) may yield immediate benefits (10). Such interventions, with their low reliance on specialized psychological resources and high practicality, are well-suited for integration into the foundational psychological care within the ICU. Secondly, the study indicates that the intervention’s impact might diminish over time. This phenomenon may stem from a shift in the sources of patient anxiety after ICU discharge—moving from acute physiological stress and environmental fear during the critical illness phase to chronic worries about functional recovery, sequelae, and the future during convalescence (42). This underscores the urgent need for developing stepped or continuous psychological support protocols to ensure long-term maintenance of treatment effects (43).

### 4.3 Effect of psychological interventions on patients’ depression

This study indicates that psychological interventions may provide a slight to moderate reduction in the immediate depressive symptoms experienced by ICU patients. However, the finding is tempered by substantial heterogeneity (*I*^2^ = 86%) and a lack of sustained benefit in the short term. Subgroup analysis identified statistically significant variations attributable to factors including follow-up duration, type of intervention, mode of delivery, ICU setting, assessment instruments, and the professional expertise of the providers. Although the results of these subgroup studies are exploratory and are limited by the number and scale of the included trials, they provide crucial guidance for clinical decision-making. For instance, the environment of the ICU as a modulating factor indicates that the psychological intervention may need to be tailored according to the specific stressors and rehabilitation trajectories of different patient groups. The marked reduction in the observed effect during the short-term follow-up, coupled with the duration of follow-up as a critical influencing factor, underscores a significant challenge confronting the clinical field: the inadequacy of relying exclusively on intervention measures provided by the ICU to achieve long-term psychological health recovery in ICU survivors (44). Symptoms presenting shortly after ICU admission may diminish over time, whereas those manifesting during extended follow-up are likely to persist (45). Consequently, clinical practice must transition from offering isolated interventions to developing comprehensive care pathways. Recent systematic reviews and meta-analyses have also emphasized the necessity of the PICS follow-up system (46).

### 4.4 Effect of psychological interventions on patients’ sleep quality

This study indicates that psychological interventions do not have a statistically significant effect on improving sleep quality among ICU patients (*p* = 0.19). Nonetheless, the notably high heterogeneity observed across the studies (*I*^2^ = 97%) implies that the effect of the intervention may be influenced by a variety of factors. The sensitivity analysis indicated that the exclusion of two studies, both categorized under CBT (Ghelasi et al. (8); Li et al. (31)), resulted in a substantial reduction in overall heterogeneity, decreasing from 97 to 59%. However, the heterogeneity remained relatively elevated. This preliminary observation suggests that these two particular CBT studies are the primary contributors to the observed inconsistency. The subgroup analysis indicates that the type of intervention may play a crucial role in modulating the observed effects. Specifically, the CBT subgroup, as examined in two studies, demonstrated significant and substantial improvements in sleep. Nonetheless, this subgroup exhibited extremely high heterogeneity (*I*^2^ = 97%), and the limited number of studies precludes the ability to draw definitive conclusions.

### 4.5 Effect of psychological interventions on patients’ PTSD

This study indicated that the overall effect of psychological intervention measures on alleviating post-traumatic stress disorder among patients in the intensive care unit did not achieve statistical significance, both in terms of immediate effects (*p* = 0.54) and follow-up effects (*p* = 0.56). Upon exclusion of the study conducted by Liang et al. (29) from the sensitivity analysis, the heterogeneity index (*I*^2^) was found to be 0%. Although the combined effect size exhibited a slight alteration, it did not achieve statistical significance (*p* = 0.22). This finding suggests that the overall result, indicating ineffectiveness, is robust and not solely influenced by individual studies. Although subgroup analyses suggest that the assessment tool, follow-up duration, and ICU type may be important moderating factors, these findings are primarily based on a single study (29). They are statistically underpowered and carry the risk of multiple comparisons. Therefore, they can only serve as hypotheses for future research and should not inform clinical decision-making.

### 4.6 Effect of psychological interventions on patients’ quality of life

The results of our meta-analysis indicated that psychological interventions did not lead to a statistically significant enhancement in patients’ quality of life, either immediately post-intervention (*p* = 0.14) or during the short-term follow-up period (*p* = 0.57). The sensitivity analysis indicated that the exclusion of the study conducted by Li et al. (31) resulted in the complete elimination of heterogeneity (*I*^2^ = 0%); however, the overall effect remained statistically insignificant. This finding suggests that the overall conclusion is robust and not unduly influenced by any single study. This indicates that, while the psychological interventions are deemed safe, their impact on the comprehensive and multifaceted construct of quality of life remains constrained. Quality of life is a multifaceted construct that encompasses elements from the physical, psychological, and social domains (47).

### 4.7 Effect of psychological interventions on the families of ICU patients

Despite some preliminary evidence suggesting potential benefits of specific interventions in selected outcomes, the aggregated results of this meta-analysis did not show statistically significant effects. The meta-analysis examining symptoms of anxiety, depression, and post-traumatic stress disorder revealed no statistically significant differences between the intervention and control groups at both the immediate post-intervention and short-term follow-up assessments. However, considerable and at times substantial heterogeneity was observed in the aggregated estimates; for example, the *I*^2^ statistic for the immediate effect of anxiety reached as high as 79%, while that for PTSD was 65%. The sensitivity analysis suggests that the observed heterogeneity in post-traumatic stress disorder outcomes is predominantly attributable to the study conducted by Nielsen et al. (37). The reason might be related to the cross-contamination between the intervention group and the control group in the study. Concerning the quality of life, the limited availability of data precludes the possibility of conducting a quantitative synthesis. Furthermore, the individual studies that are accessible do not indicate any significant differences between the groups (32, 33, 37).

The considerable heterogeneity observed may be attributable to the varying baseline vulnerabilities and pre-existing mental health risk factors among family members, which the included trials may not have sufficiently accounted for. Family members of ICU patients experience psychological stress not just from the sudden ICU admission, but also from the ‘cumulative burden’ or ‘double blow’ effect due to existing stressors piling up. For instance, the family members’ personal history of severe infections (such as COVID-19) can exacerbate the psychological health problems of the ICU patients’ families (48). Moreover, sociodemographic and role-related factors, including gender—wherein women generally report elevated levels of distress—occupational stress, and the substantial caregiving responsibilities assumed during or prior to an ICU stay, can markedly elevate the baseline psychological risk (49). In conclusion, the present findings, which are statistically insignificant, should not be construed as definitive evidence that psychological support is ineffective for all family members of patients in the ICU.

### 4.8 Strengths and limitations

This study possesses several strengths: the reporting strictly adhered to the PRISMA guidelines, a comprehensive search strategy was implemented to minimize omissions, and both patients and their family members—two crucial populations—were examined. However, the study also presents limitations: (1) The number of included studies was limited, particularly concerning outcomes related to family members and certain secondary outcomes, thereby constraining the certainty of the conclusions. (2) Substantial heterogeneity was observed in several pooled analyses. Although we performed sensitivity analyses and extensive subgroup analyses, some potential sources of heterogeneity—such as specific intervention intensity and detailed patient acuity—could not be fully explored due to inconsistent reporting in the original studies. (3) Exploratory results of subgroup analysis: the number of studies included in most subgroup analyses is limited, and the statistical test power may be insufficient. These results are meant to generate hypotheses, not conclusions, and should be interpreted with caution. (4) Additionally, the funnel plot analysis revealed asymmetry in depression, sleep quality, and PTSD outcomes, suggesting the possibility of publication bias.

## 5 Conclusion

In conclusion, this meta-analysis offers a comprehensive assessment of the effectiveness of psychological interventions for patients in intensive care units and their family members. Preliminary evidence suggests that specific types of interventions (such as those based on cognitive behavioral therapy or mindfulness) may show potential in improving specific outcomes such as anxiety and depression on patients. However, most of the existing evidence is based on a limited number of trials with small sample sizes, and the effects of different interventions vary significantly. Therefore, at present, no clear conclusion can be drawn regarding the general effectiveness of psychological treatment. Current evidence for psychological interventions targeting PICS-F is very limited and that further, larger randomized trials are needed. Future research should prioritize high-quality, large-sample RCTs with clearly defined and comparable interventions to verify the effectiveness of specific psychological intervention models for ICU populations.

## Data Availability

The original contributions presented in the study are included in the article/Supplementary material, further inquiries can be directed to the corresponding authors.
