# Supplementary material for: Effects of psychological intervention on outcomes of critically ill patients and their families: a systematic review and meta-analysis
Source: Front Med (Lausanne). 2026 Feb 9;13:1739015. doi: 10.3389/fmed.2026.1739015 (PMC12926472; doi:10.3389/fmed.2026.1739015)
Supplement: Supplementary file 1 [file Data_Sheet_1.docx]

**Supplementary 1. Search strategies and results for all databases.**

| **Database** | **Step** | **Search Strategy** | **Results** | **Retrieval Time** |
| --- | --- | --- | --- | --- |
| Web of Science | #1 | TS=("Psychotherapy" OR "Cognitive Behavioral Therapy" OR "Dignity Therapy" OR "Intervention, Psychosocial" OR "Interventions, Psychosocial" OR "Psychosocial Intervention*" OR "Intervention, Psychological" OR "Interventions, Psychological" OR "Aromatherapy" OR "Behavior Therapy" OR "Emotion-Focused Therapy" OR "Music Therapy" OR "Narrative Therapy" OR "Person-Centered Psychotherapy" OR "Relaxation" OR "Stress Management" OR "Counselling" OR " ICU Diary") | 963 | 02/12/2025 |
|  | #2 | TS=("ICU" OR "Intensive Care Unit*" OR "intensive care unit*" OR "intensive care department" OR "critically ill" OR "critical illness" OR "critical illnesses" OR "critical care" OR "Critical Care Unit" OR "high dependency unit") |  |  |
|  | #3 | TS=("Controlled Clinical Trials" OR "Randomized Controlled Trials" OR "RCT*" OR "Intention to Treat Analysis" OR "Pragmatic Clinical Trials" OR "Single-Blind Method" OR "Double-Blind Method" OR "Cohort Studies" OR "Case-Control Studies" OR "Cross-Sectional Studies" OR "Longitudinal Studies" OR "random*" OR "blind*" OR "singleblind*" OR "doubleblind*" OR "tripleblind*") |  |  |
|  | #4 | #1 AND #2 AND #3 |  |  |
| Embase | #1 | 'psychotherapy':ab,ti OR 'psychotherapy':ab,ti OR 'cognitive behavioral therapy':ab,ti OR 'cognitive behavioral therapy':ab,ti OR 'dignity therapy':ab,ti OR 'dignity therapy':ab,ti OR 'intervention, psychosocial':ab,ti OR 'interventions, psychosocial':ab,ti OR 'psychosocial intervention':ab,ti OR 'psychosocial intervention':ab,ti OR 'intervention, psychological':ab,ti OR 'interventions, psychological':ab,ti OR 'aromatherapy':ab,ti OR 'aromatherapy':ab,ti OR 'behavior therapy':ab,ti OR 'behavior therapy':ab,ti OR 'emotion-focused therapy':ab,ti OR 'emotion-focused therapy':ab,ti OR 'music therapy':ab,ti OR 'music therapy':ab,ti OR 'narrative therapy':ab,ti OR 'narrative therapy':ab,ti OR 'person-centered psychotherapy':ab,ti OR 'person-centered psychotherapy':ab,ti OR 'relaxation':ab,ti OR 'relaxation':ab,ti OR 'stress management':ab,ti OR 'stress management':ab,ti OR 'counselling':ab,ti OR 'counselling':ab,ti OR ' ICU Diary ':ab,ti | 685 | 02/12/2025 |
|  | #2 | 'icu':ab,ti OR 'intensive care unit':ab,ti OR 'intensive care units':ab,ti OR 'intensive care department':ab,ti OR 'critically ill':ab,ti OR 'critical illness':ab,ti OR 'critical illnesses':ab,ti OR 'critical care':ab,ti OR 'critical care unit':ab,ti OR 'high dependency unit':ab,ti |  |  |
|  | #3 | 'controlled clinical trials' OR 'randomized controlled trials' OR 'rct*' OR 'intention to treat analysis' OR 'pragmatic clinical trials' OR 'single-blind method' OR 'double-blind method' OR 'cohort studies' OR 'case-control studies' OR 'cross-sectional studies' OR 'longitudinal studies' OR 'random*' OR 'blind*' OR 'singleblind*' OR 'doubleblind*' OR 'tripleblind*' |  |  |
|  | #4 | #1 AND #2 AND #3 |  |  |
| Cochrane Library | #1 | （"Psychotherapy" OR "Cognitive Behavioral Therapy" OR "Dignity Therapy" OR "Intervention, Psychosocial" OR "Interventions, Psychosocial" OR "Psychosocial Intervention" OR "Psychosocial Intervention" OR "Intervention, Psychological" OR "Interventions, Psychological" OR "Aromatherapy" OR "Behavior Therapy" OR "Emotion-Focused Therapy" OR "Music Therapy" OR "Narrative Therapy" OR "Person-Centered Psychotherapy" OR "Relaxation" OR "Stress Management" OR "Counselling" OR " ICU Diary "）:ti,ab,kw | 1761 | 02/12/2025 |
|  | #2 | （"ICU" OR "Intensive Care Unit" OR "Intensive Care Units" OR "intensive care unit" OR "intensive care units" OR "intensive care department" OR "critically ill" OR "critical illness" OR "critical illnesses" OR "critical care" OR "Critical Care Unit" OR "high dependency unit"）:ti,ab,kw |  |  |
|  | #3 | #1 AND #2 |  |  |
| PubMed | #1 | "Psychotherapy"[Mesh] OR "Cognitive Behavioral Therapy"[Mesh] OR "Dignity Therapy"[Mesh] OR "Intervention, Psychosocial"[Title/Abstract] OR "Interventions, Psychosocial"[Title/Abstract] OR "Psychosocial Intervention*"[Title/Abstract] OR "Intervention, Psychological"[Title/Abstract] OR "Interventions, Psychological"[Title/Abstract] OR "Aromatherapy"[Title/Abstract] OR "Behavior Therapy"[Title/Abstract] OR "Dignity Therapy"[Title/Abstract] OR "Emotion-Focused Therapy"[Title/Abstract] OR "Music Therapy"[Title/Abstract] OR "Narrative Therapy"[Title/Abstract] OR "Person-Centered Psychotherapy"[Title/Abstract] OR "Relaxation"[Title/Abstract] OR "Stress Management"[Title/Abstract] OR "Counselling"[Title/Abstract] OR " ICU Diary "[Title/Abstract] | 817 | 02/12/2025 |
|  | #2 | Intensive Care Unit[Mesh] OR "ICU"[Title/Abstract] OR "Intensive Care Unit*"[Title/Abstract] OR "intensive care unit*"[Title/Abstract] OR "intensive care department"[Title/Abstract] OR "critically ill"[Title/Abstract] OR "critical illness"[Title/Abstract] OR "critical illnesses"[Title/Abstract] OR "critical care"[Title/Abstract] OR "Critical Care Unit"[Title/Abstract] OR "high dependency unit"[Title/Abstract] |  |  |
|  | #3 | "Controlled Clinical Trials as Topic"[MeSH Terms] OR "Randomized Controlled Trials as Topic"[MeSH Terms] OR "Intention to Treat Analysis"[MeSH Terms] OR "Pragmatic Clinical Trials as Topic"[MeSH Terms] OR "Single-Blind Method"[MeSH Terms] OR "Double-Blind Method"[MeSH Terms] OR "Cohort Studies"[MeSH Terms] OR "Case-Control Studies"[MeSH Terms] OR "Cross-Sectional Studies"[MeSH Terms] OR "Longitudinal Studies"[MeSH Terms] OR "random*"[Title/Abstract] OR "blind*"[Title/Abstract] OR "singleblind*"[Title/Abstract] OR "doubleblind*"[Title/Abstract] OR "tripleblind*"[Title/Abstract] |  |  |
|  | #4 | #1 AND #2 AND #3 |  |  |


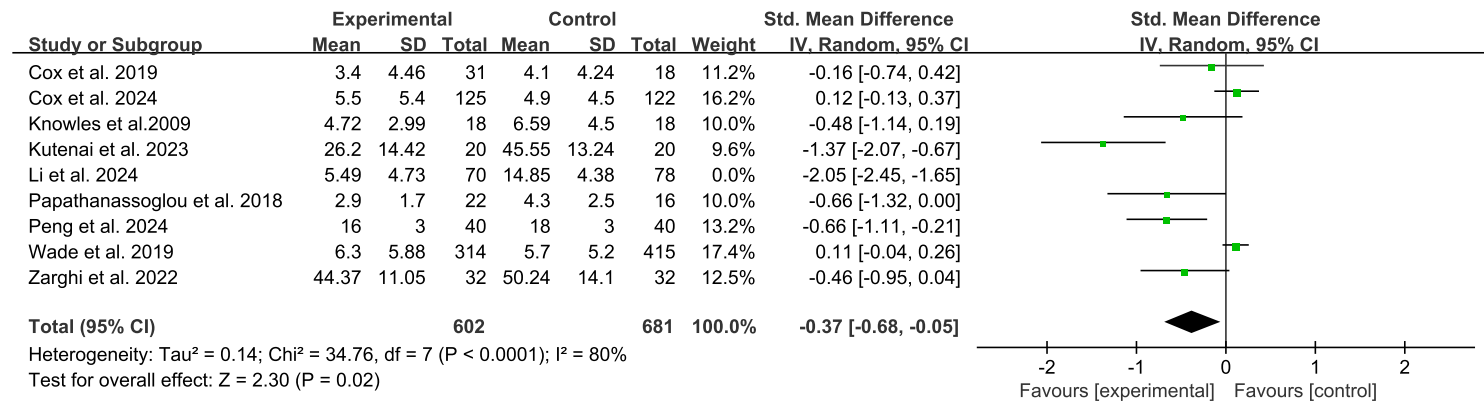
 Supplementary 2

Forest plot of the meta-analysis on patients' anxiety (post-intervention) –sensitivity analysis


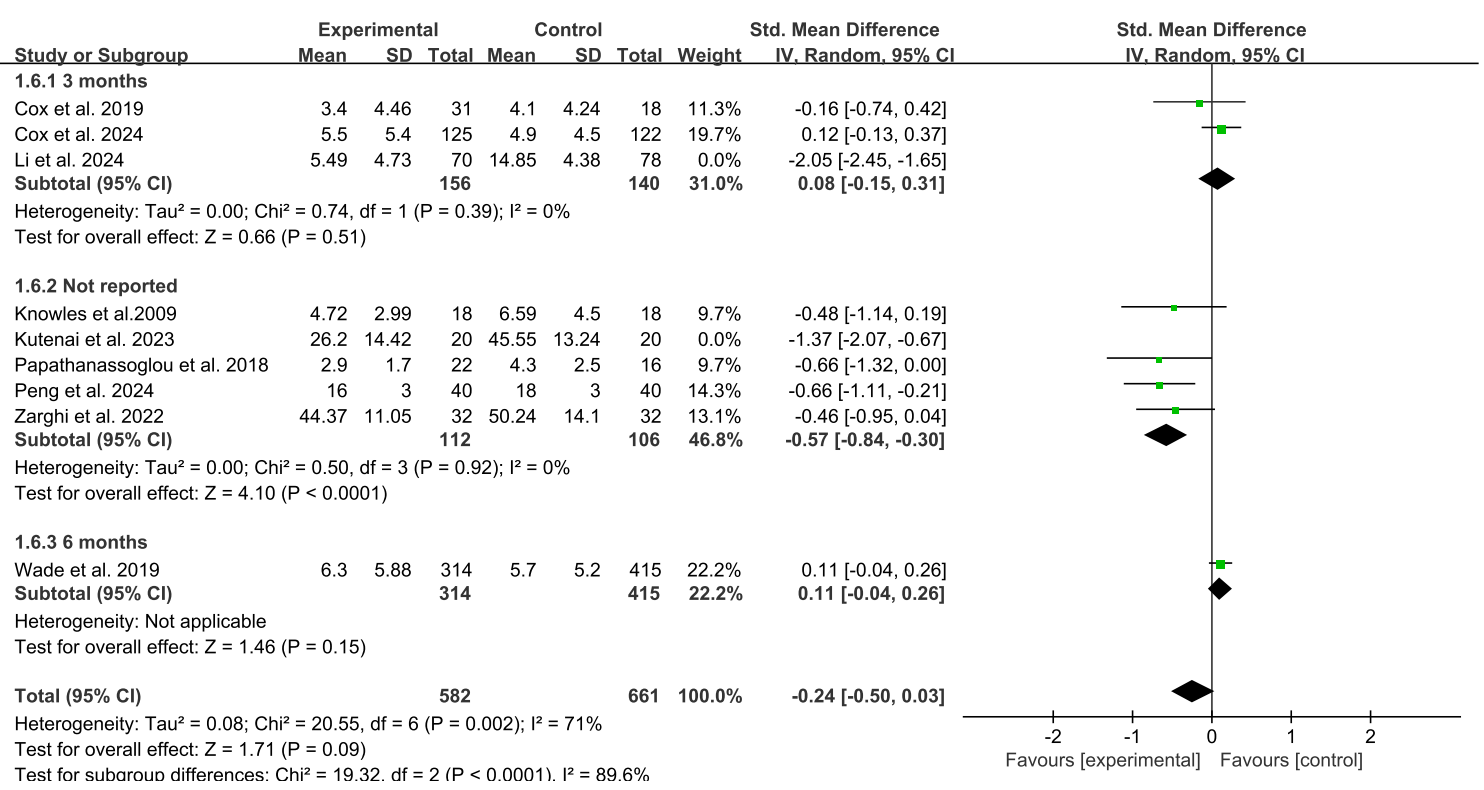


Supplementary 3

Forest plot of the meta-analysis on patients' anxiety (post-intervention) - subgroup by follow-up duration


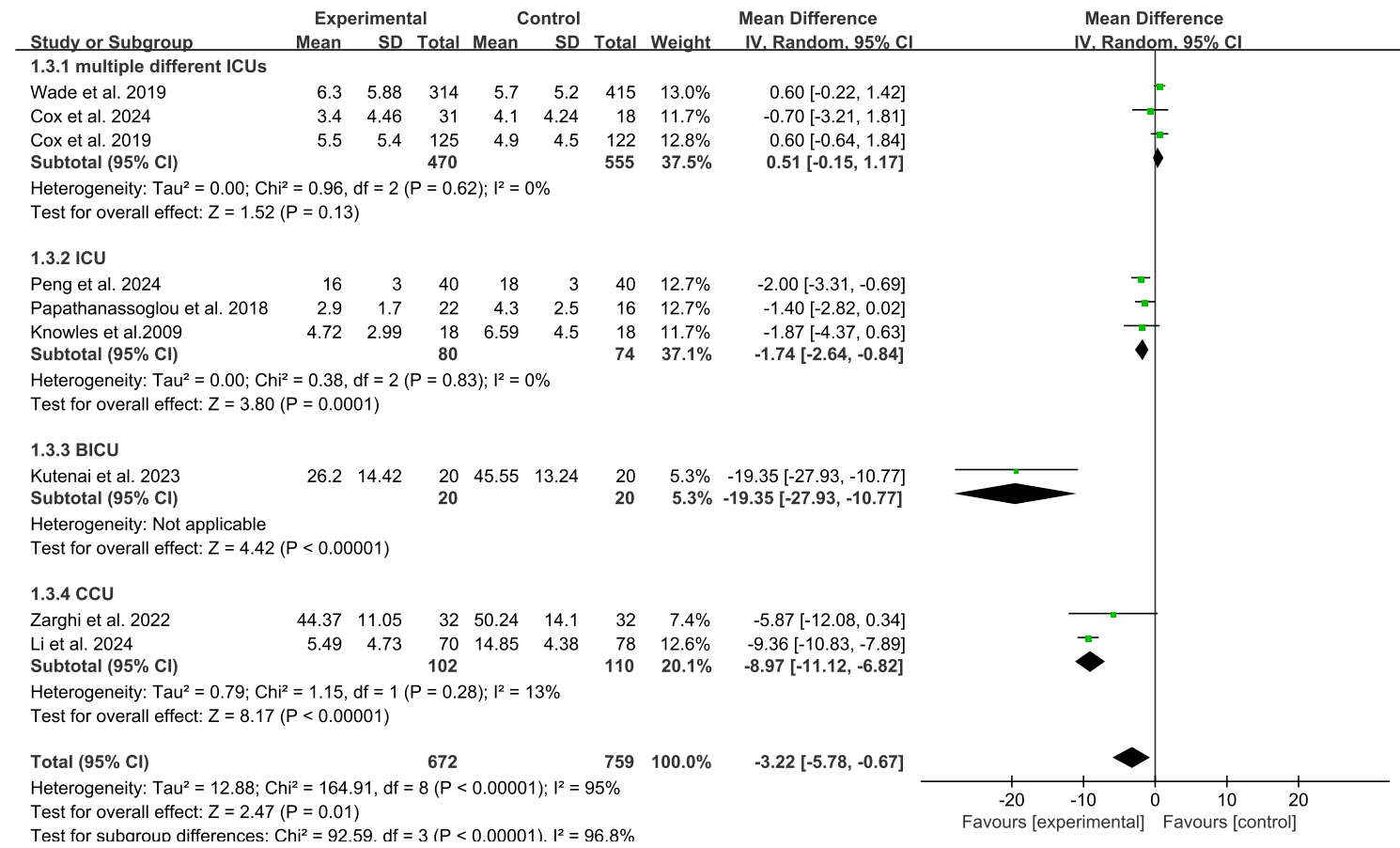


Supplementary 4

Forest plot of the meta-analysis on patients' anxiety (post-intervention) - subgroup by ICU setting


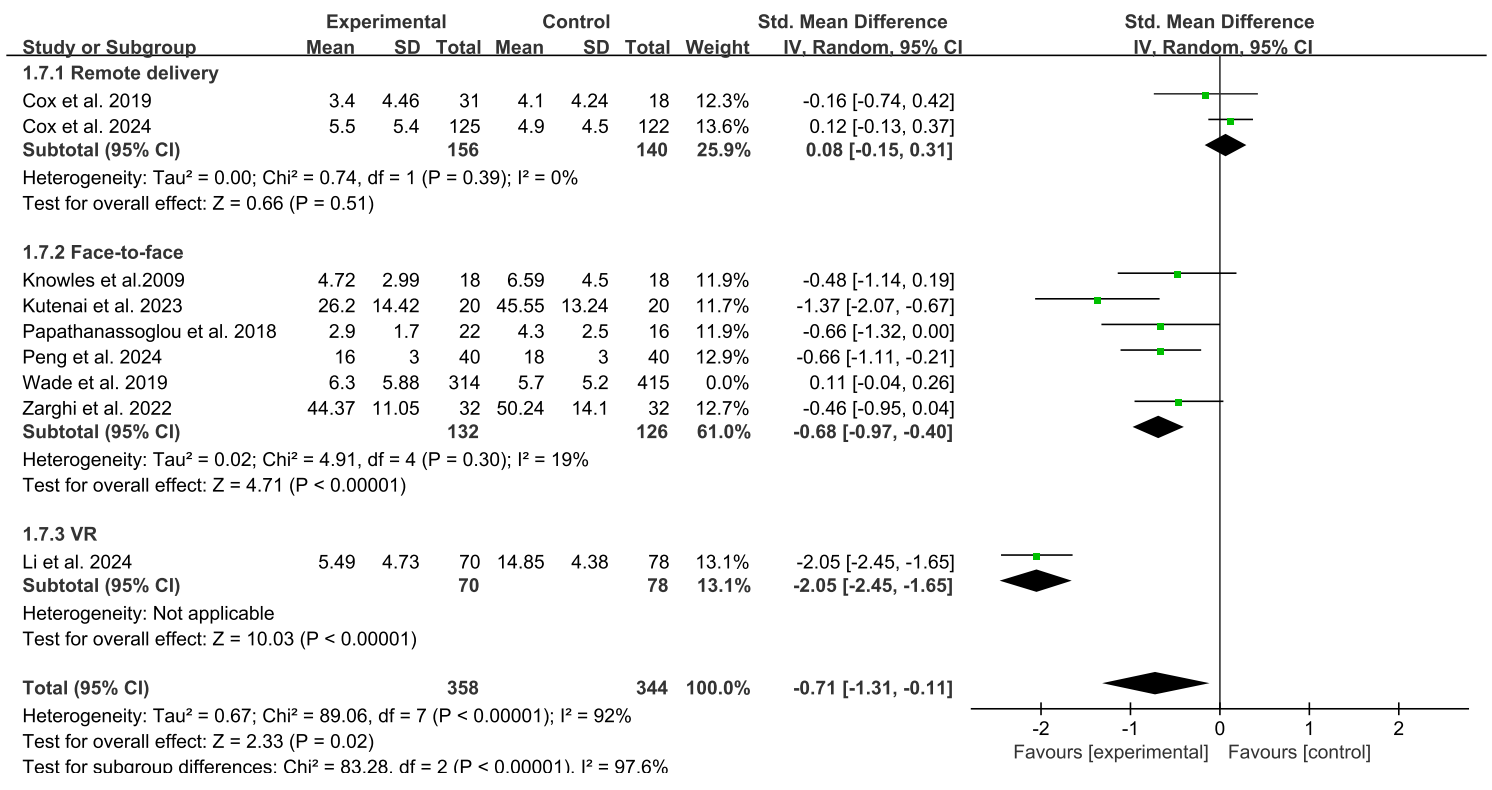


Supplementary 5

Forest plot of the meta-analysis on patients' anxiety (post-intervention) - subgroup by mode of delivery


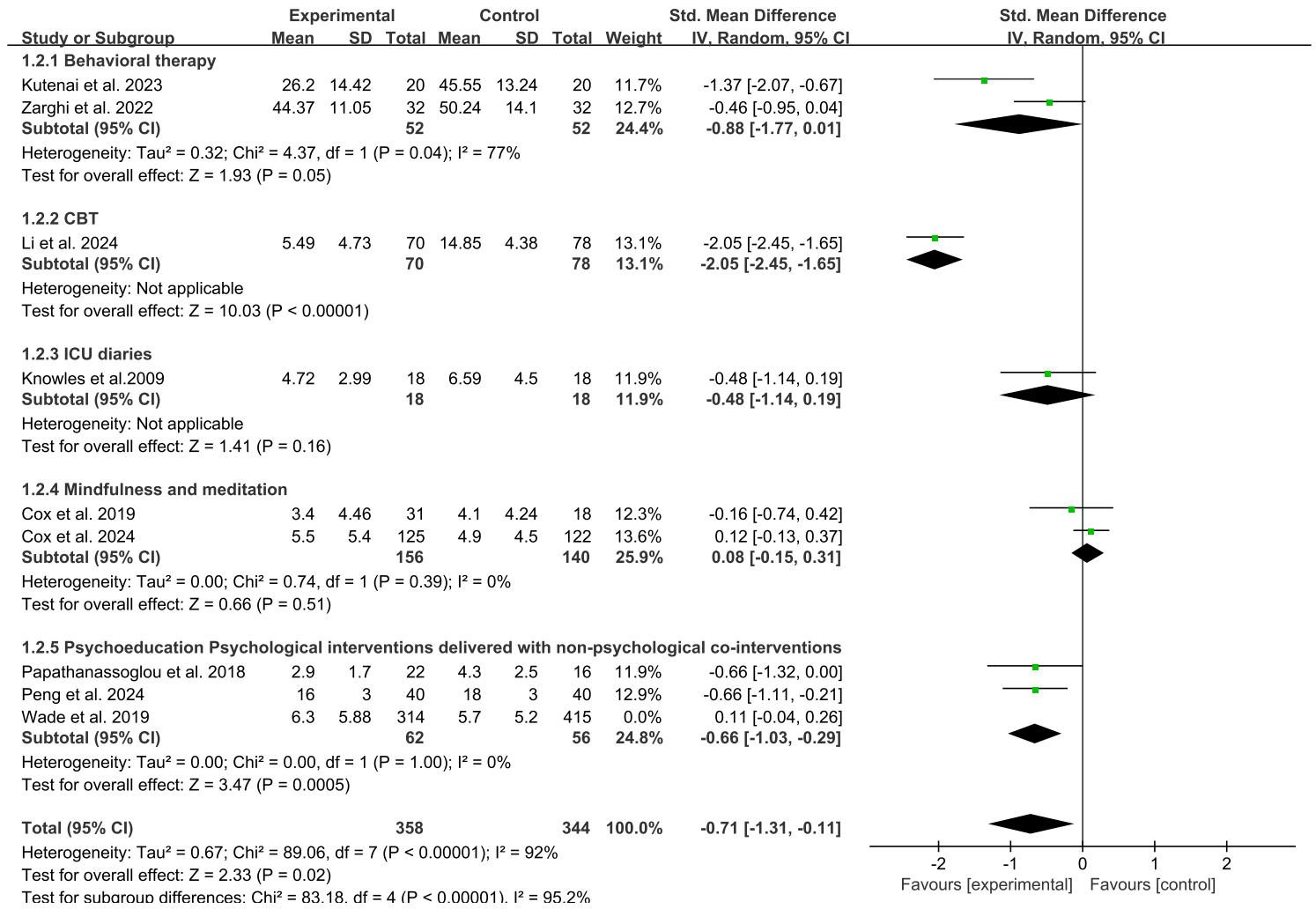


Supplementary 6

Forest plot of the meta-analysis on patients' anxiety (post-intervention) - subgroup by type of psychological intervention


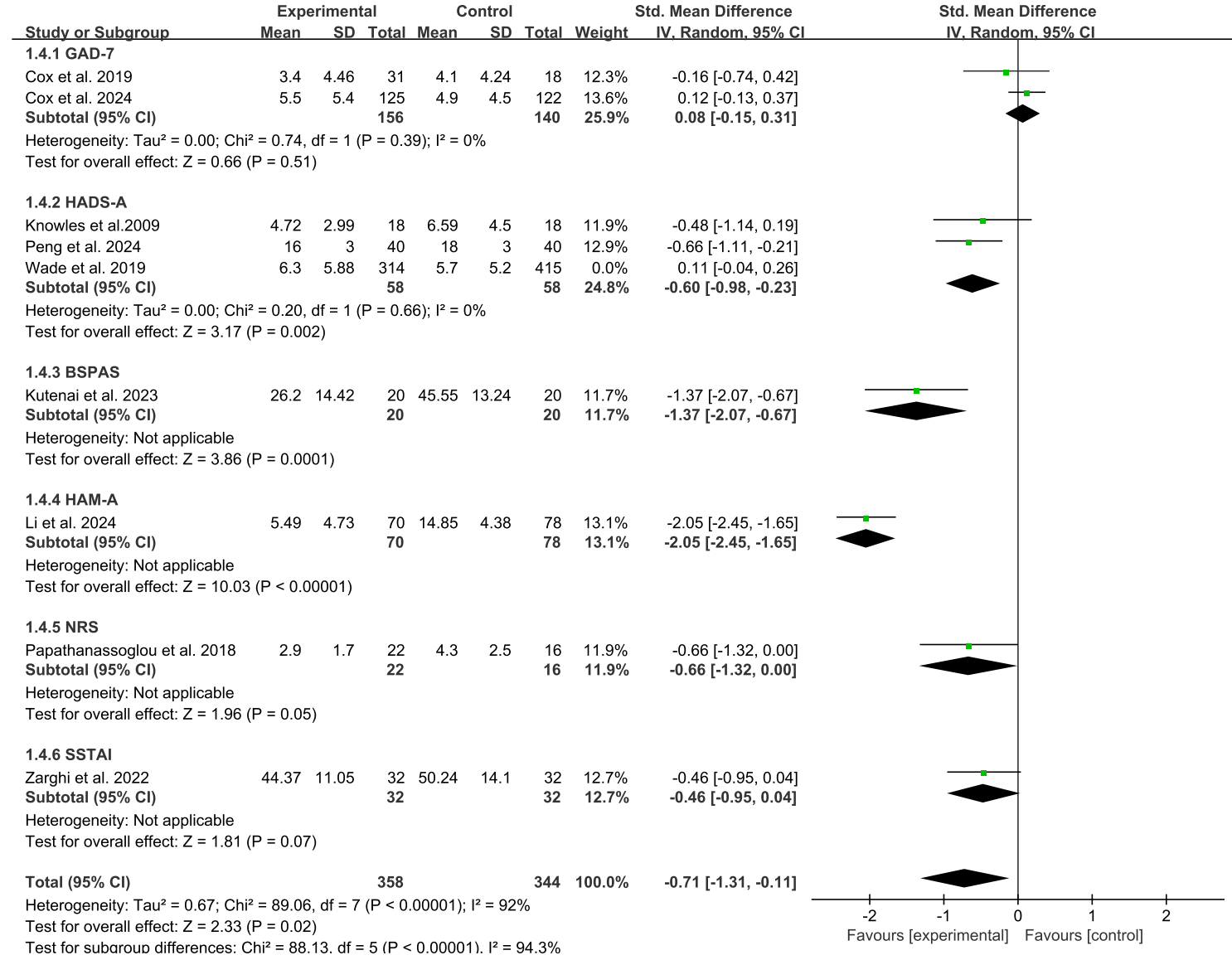


Supplementary 7

Forest plot of the meta-analysis on patients' anxiety (post-intervention) - subgroup by assessment tool


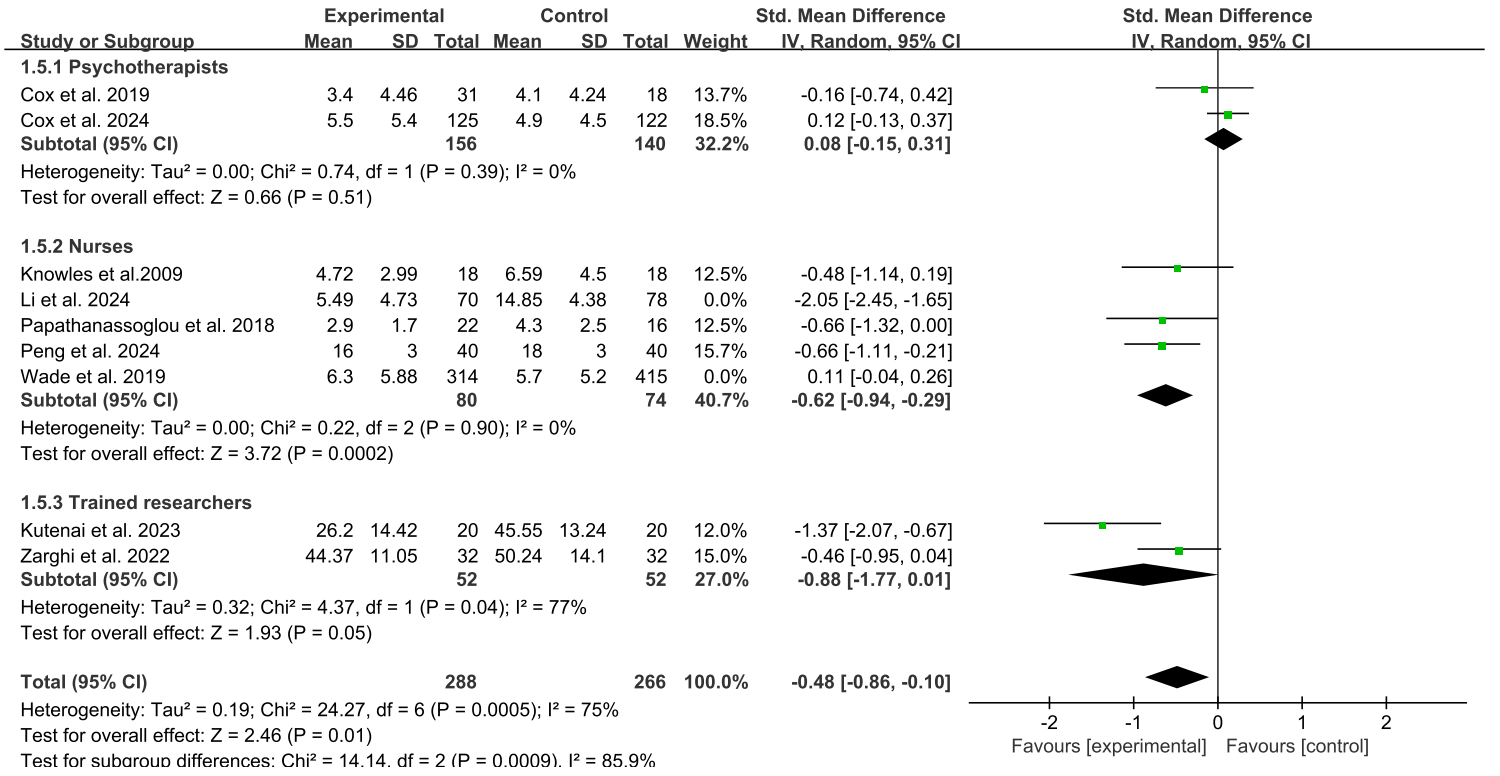
Supplementary 8

Forest plot of the meta-analysis on patients' anxiety (post-intervention) - subgroup by intervention provider


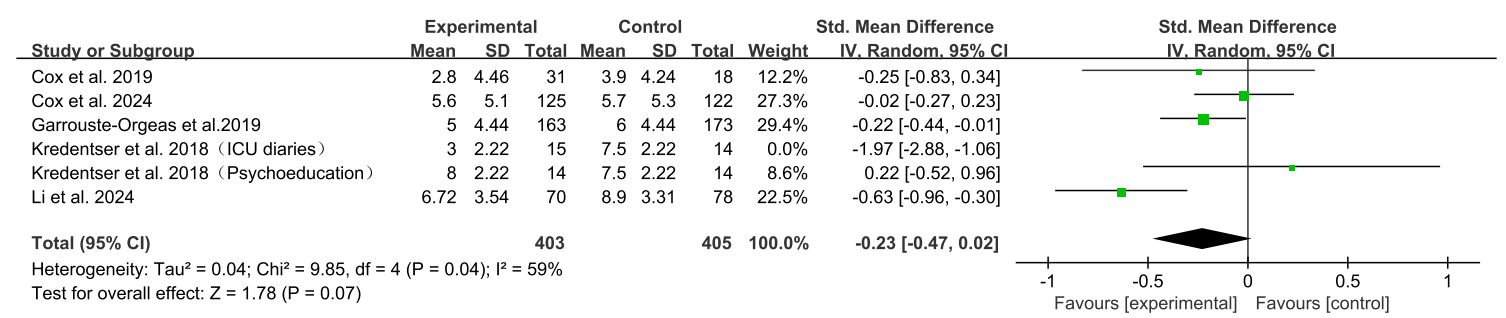


Supplementary 9

Forest plot of the meta-analysis on patients' anxiety (short-term follow-up) –sensitivity analysis


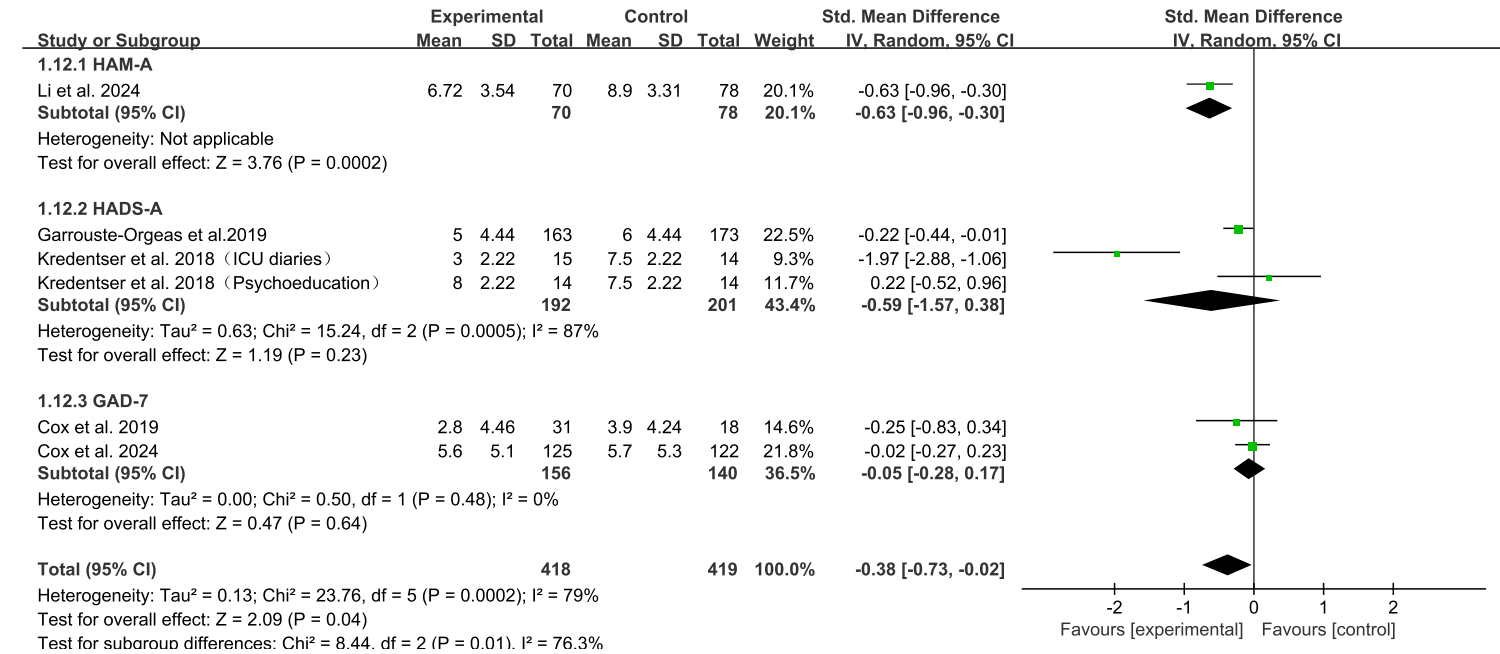
 Supplementary 10

Forest plot of the meta-analysis on patients' anxiety (short-term follow-up) –subgroup by assessment tool


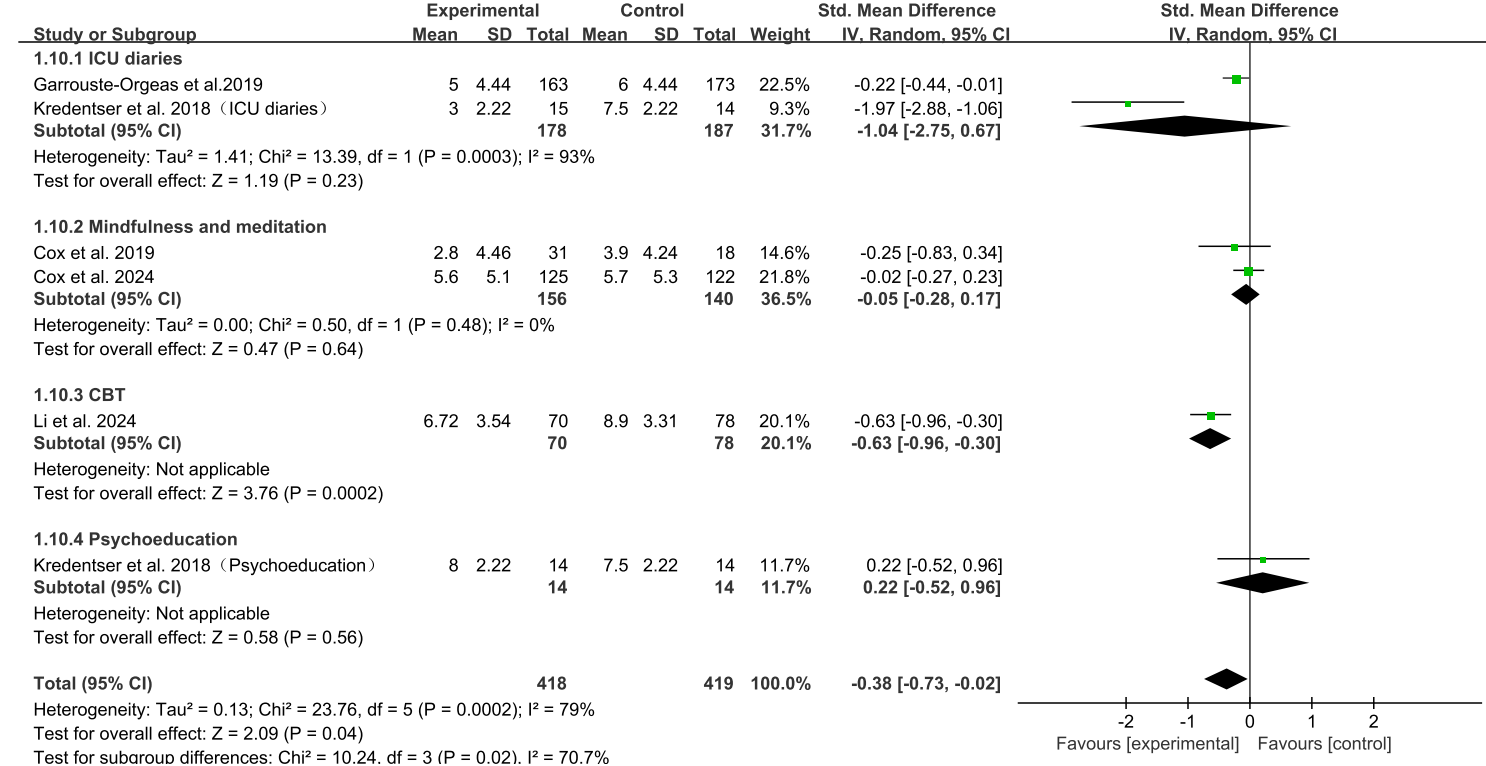
 Supplementary 11

Forest plot of the meta-analysis on patients' anxiety (short-term follow-up) –subgroup by type of psychological intervention


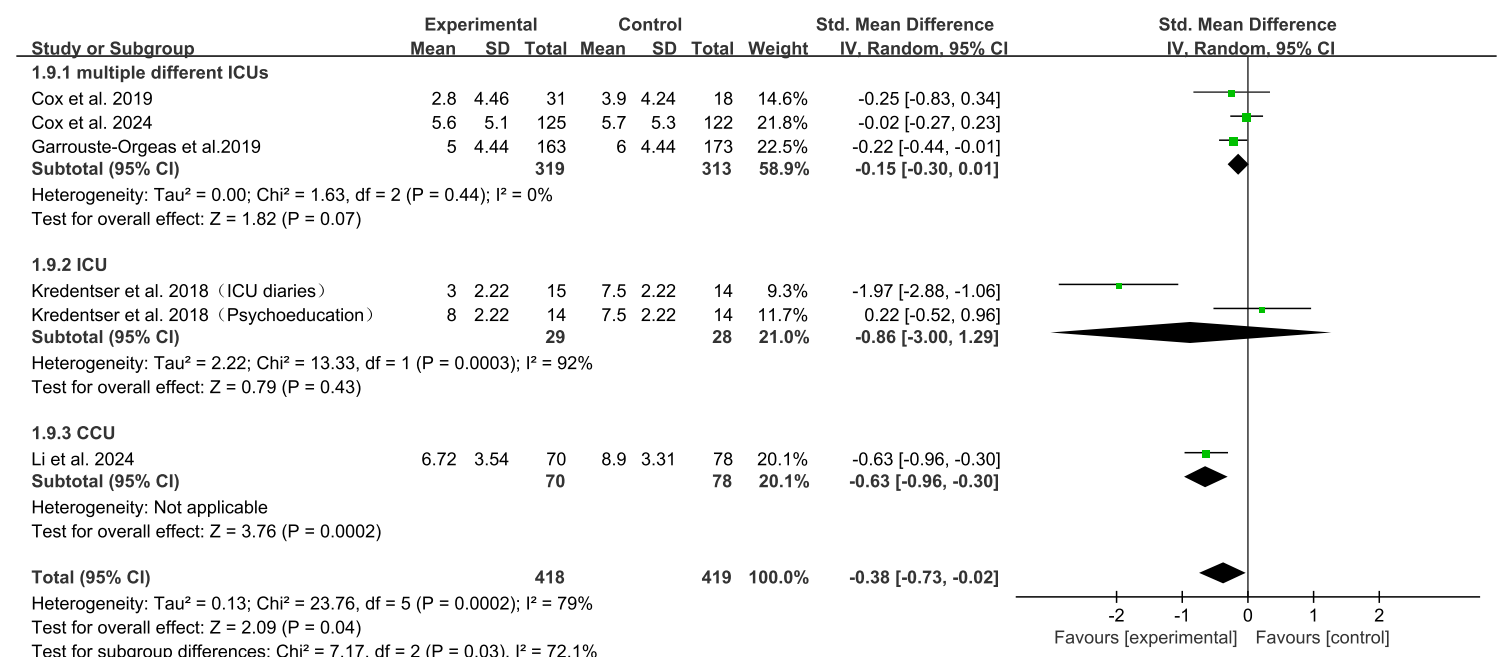
 Supplementary 12

Forest plot of the meta-analysis on patients' anxiety (short-term follow-up) –subgroup by ICU setting


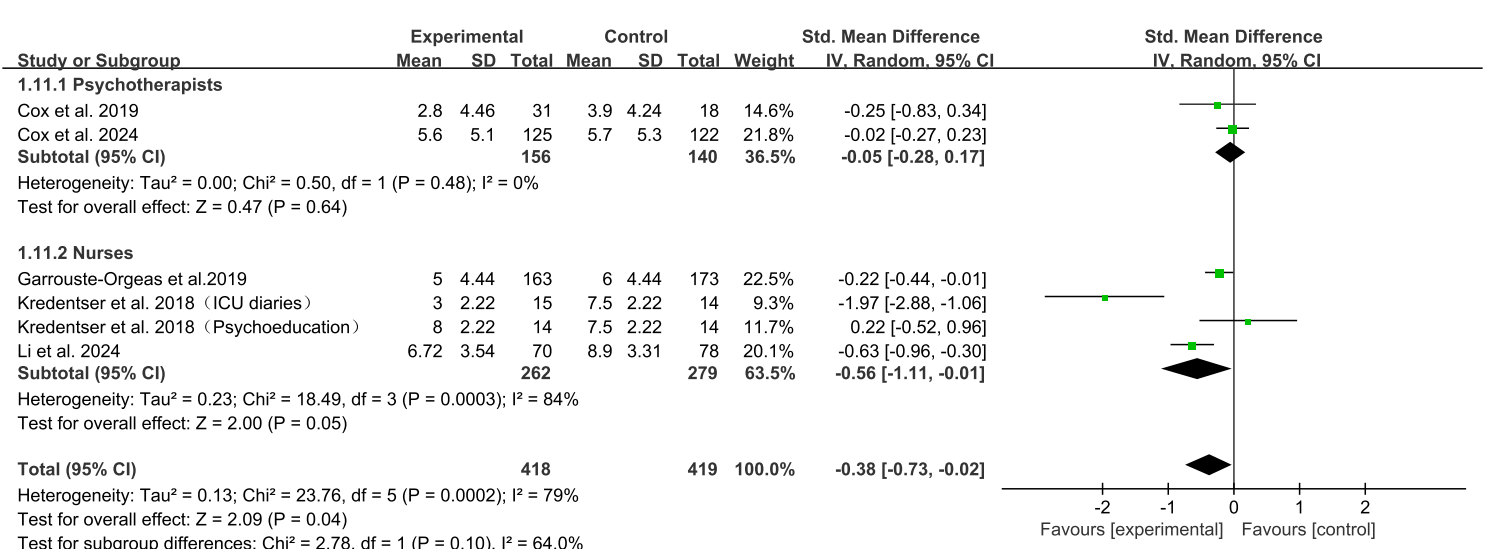
 Supplementary 13

Forest plot of the meta-analysis on patients' anxiety (short-term follow-up) –subgroup by intervention provider


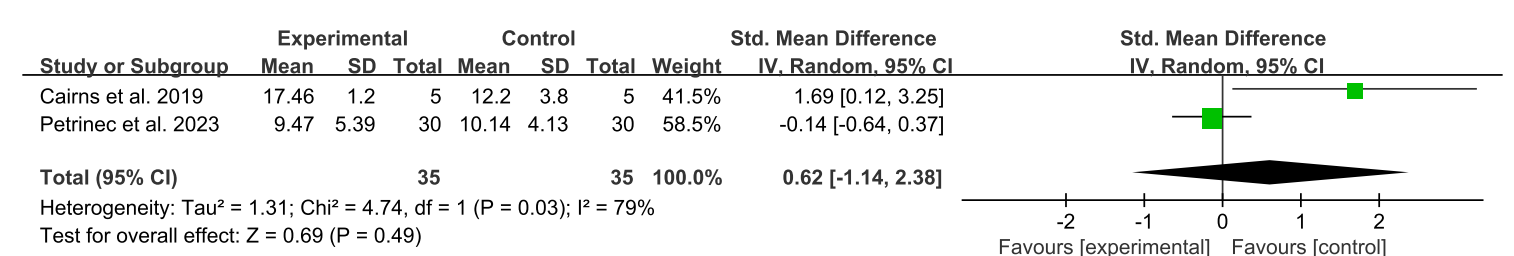
Supplementary 14

Forest plot of the meta-analysis on anxiety in family members (post-intervention)


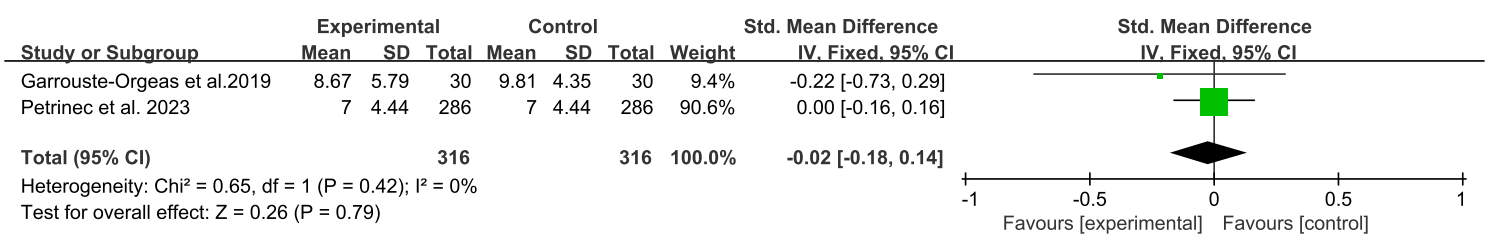
Supplementary 15

Forest plot of the meta-analysis on anxiety in family members (short-term follow-up)


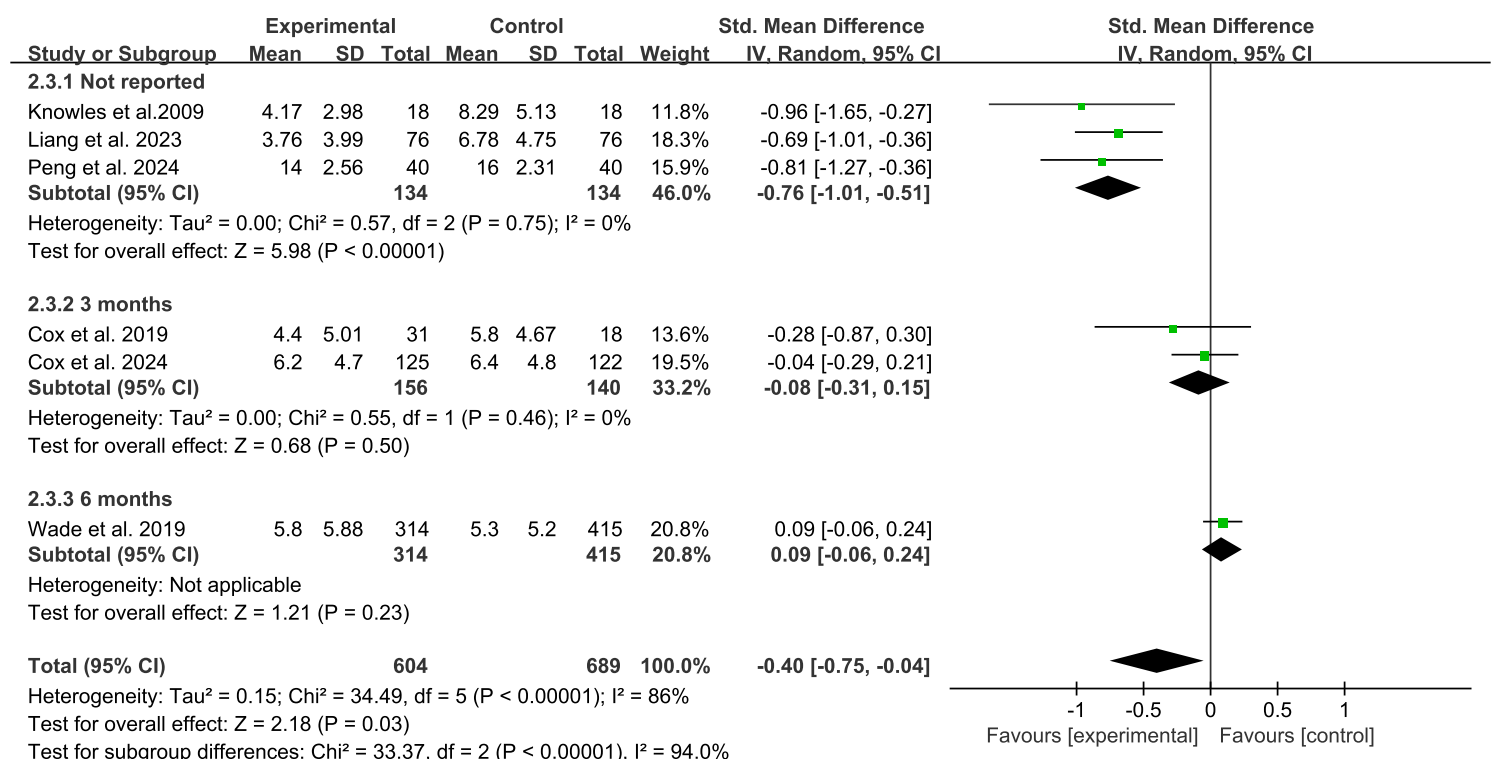
 Supplementary 16

Forest plot of the meta-analysis on patients' depression (post-intervention) - subgroup by follow-up duration


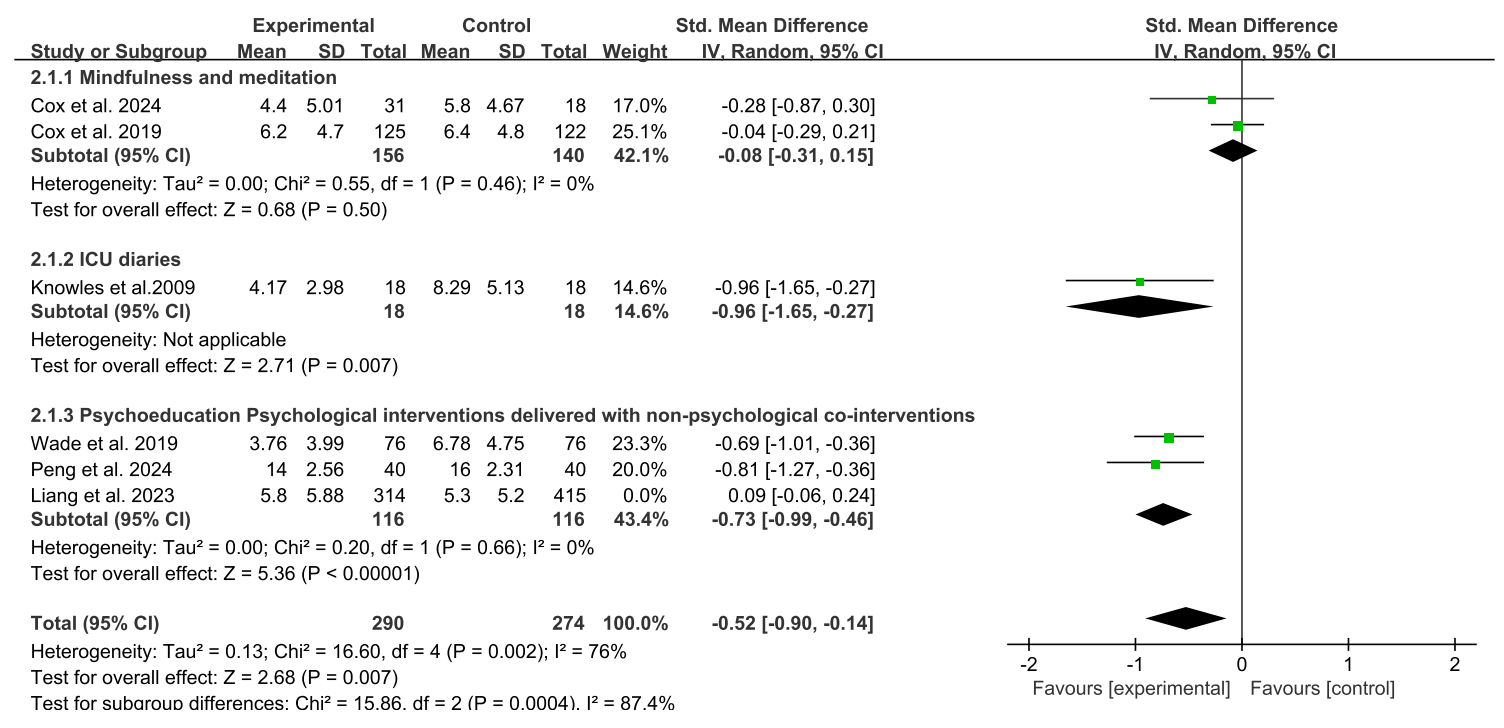
 Supplementary 17

Forest plot of the meta-analysis on patients' depression (post-intervention) - subgroup by type of psychological intervention


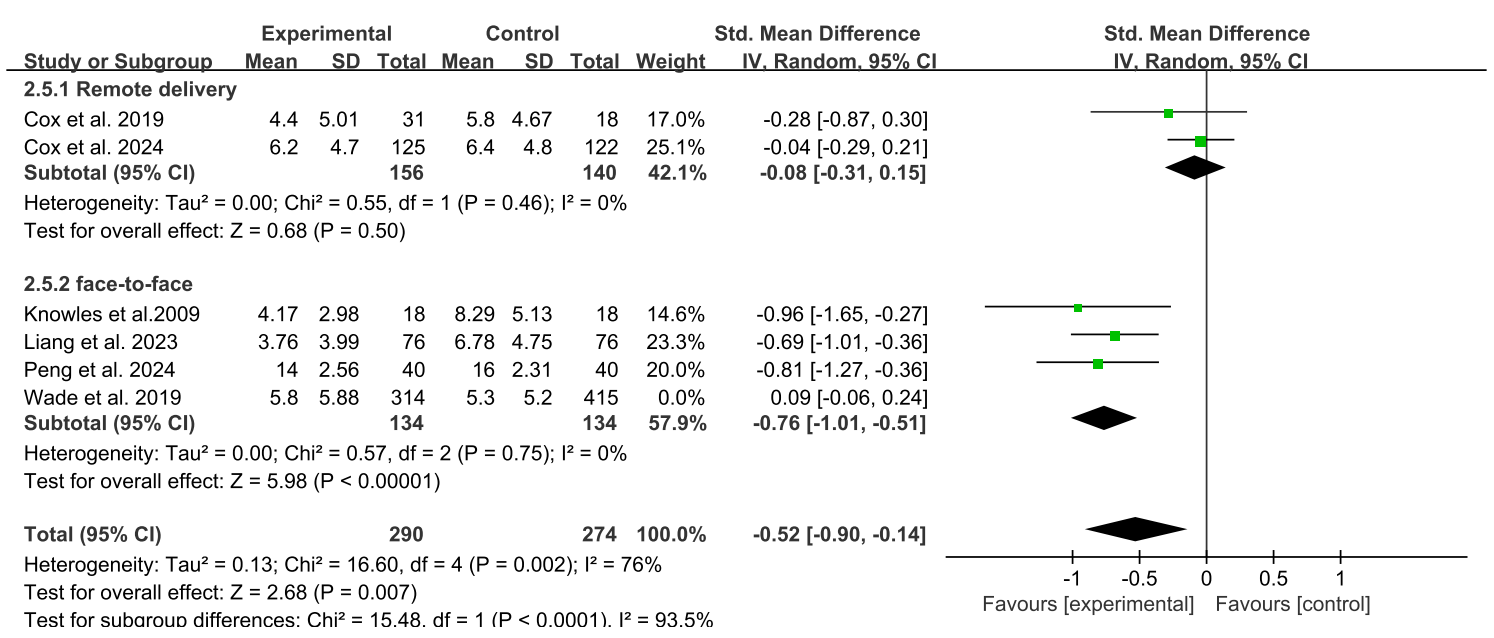
 Supplementary 18

Forest plot of the meta-analysis on patients' depression (post-intervention) - subgroup by mode of delivery


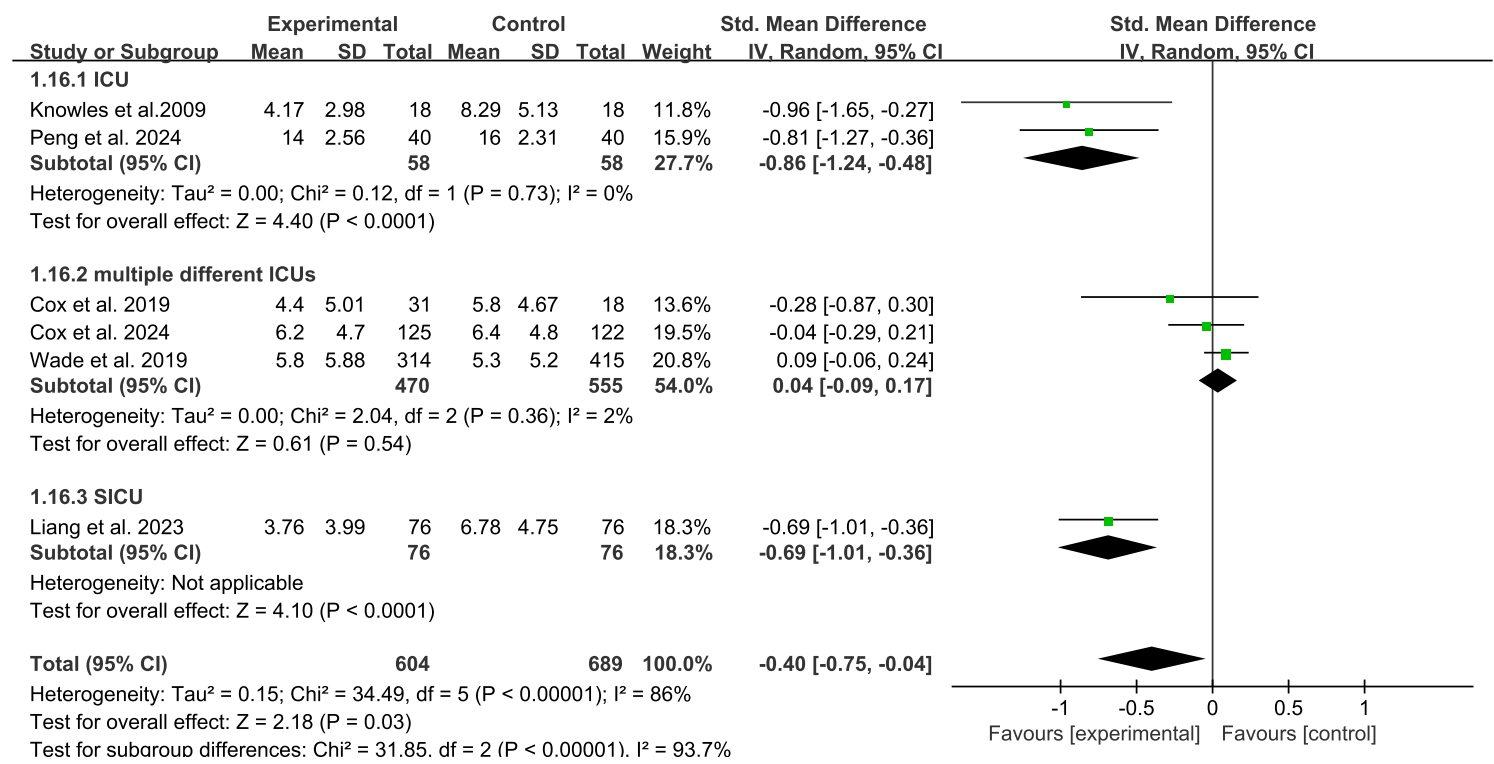
 Supplementary 19

Forest plot of the meta-analysis on patients' depression (post-intervention) - subgroup by ICU setting


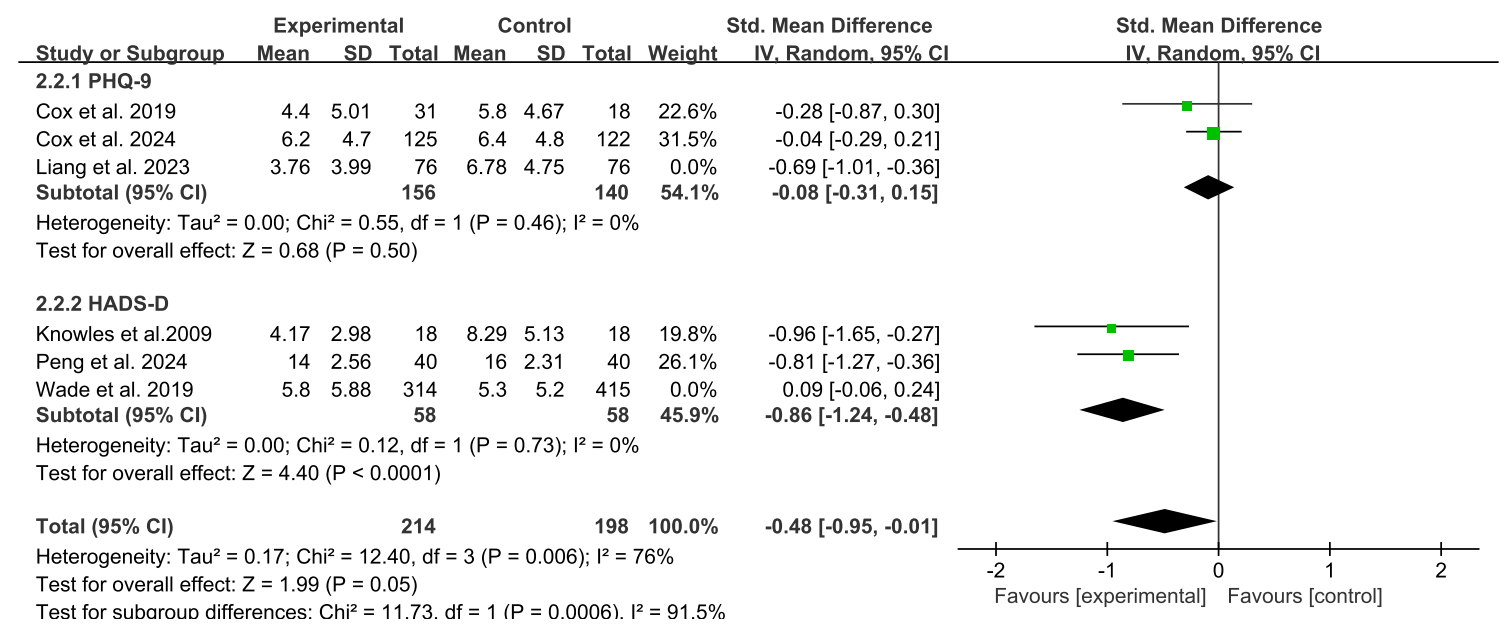


Supplementary 20

Forest plot of the meta-analysis on patients' depression (post-intervention) - subgroup by assessment tool


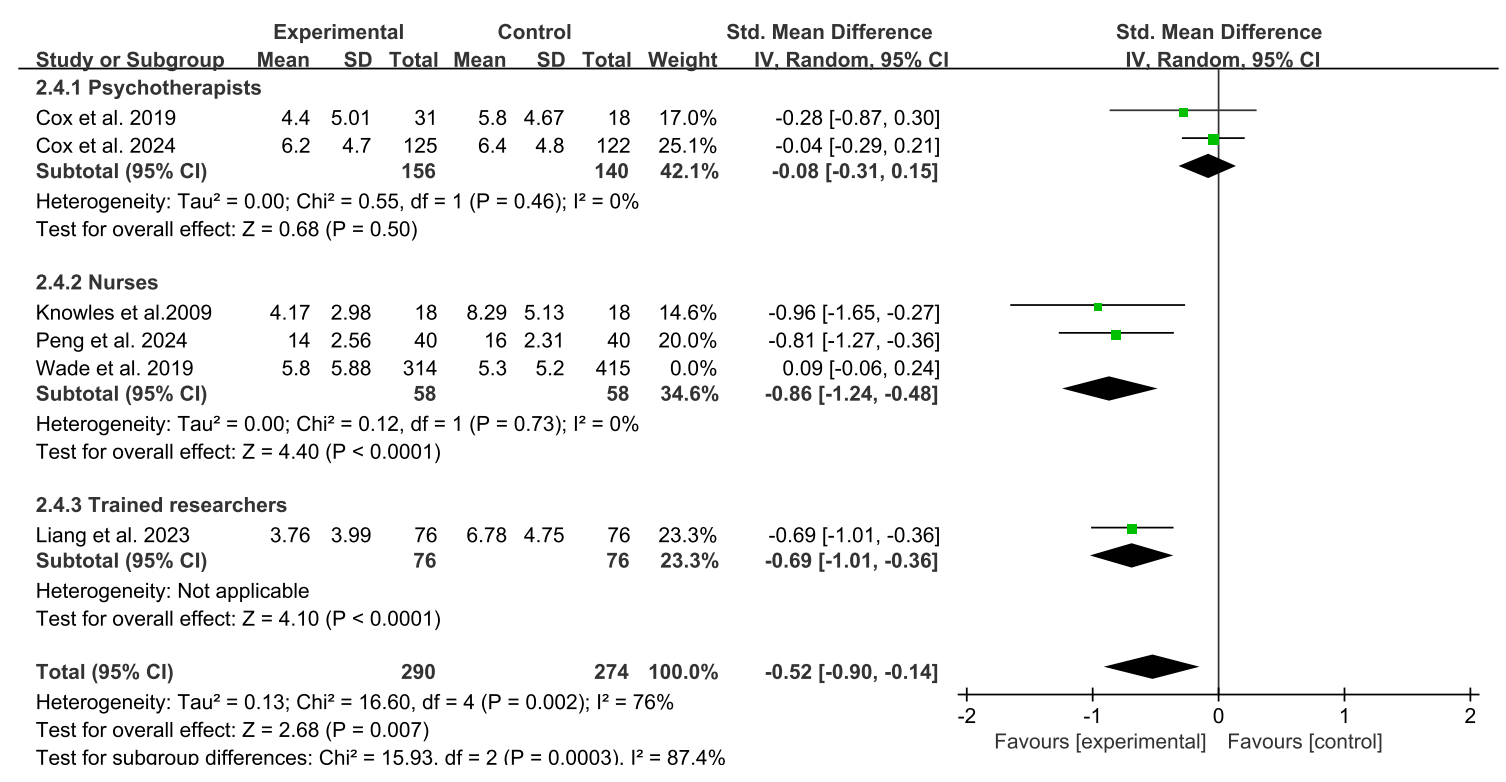
 Supplementary 21

Forest plot of the meta-analysis on patients' depression (post-intervention) - subgroup by provider expertise


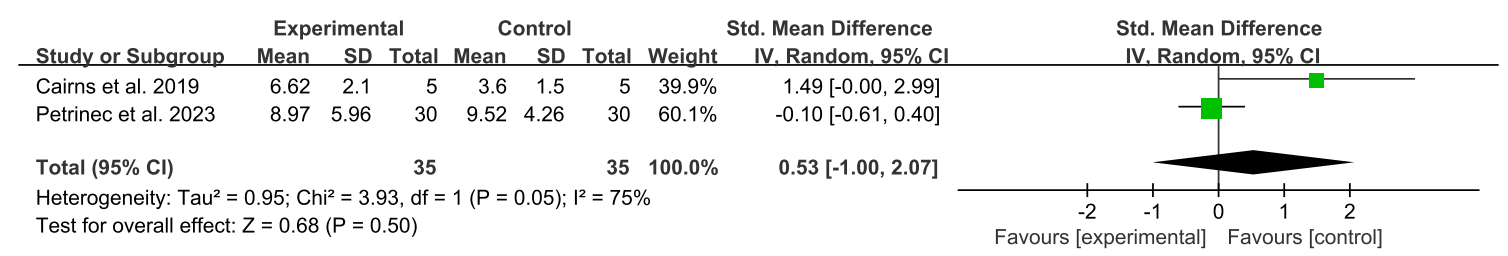


Supplementary 22

Forest plot of the meta-analysis on depression in family members (post-intervention)


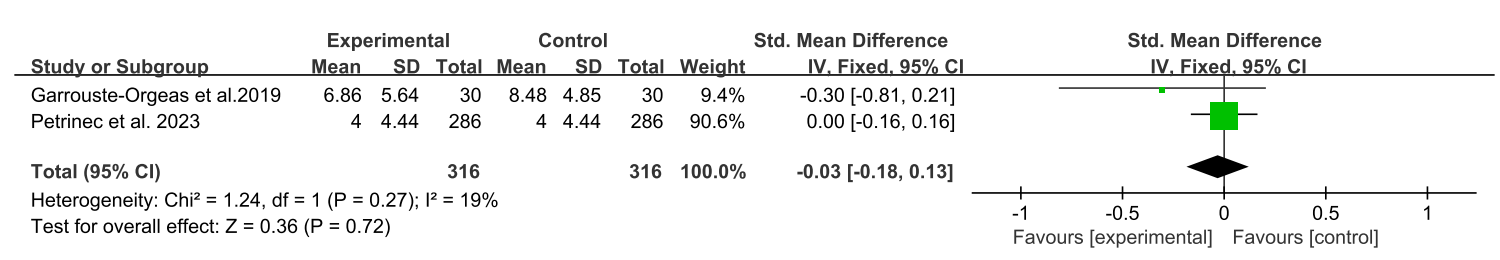
 Supplementary 23

Forest plot of the meta-analysis on depression in family members (short-term follow-up)


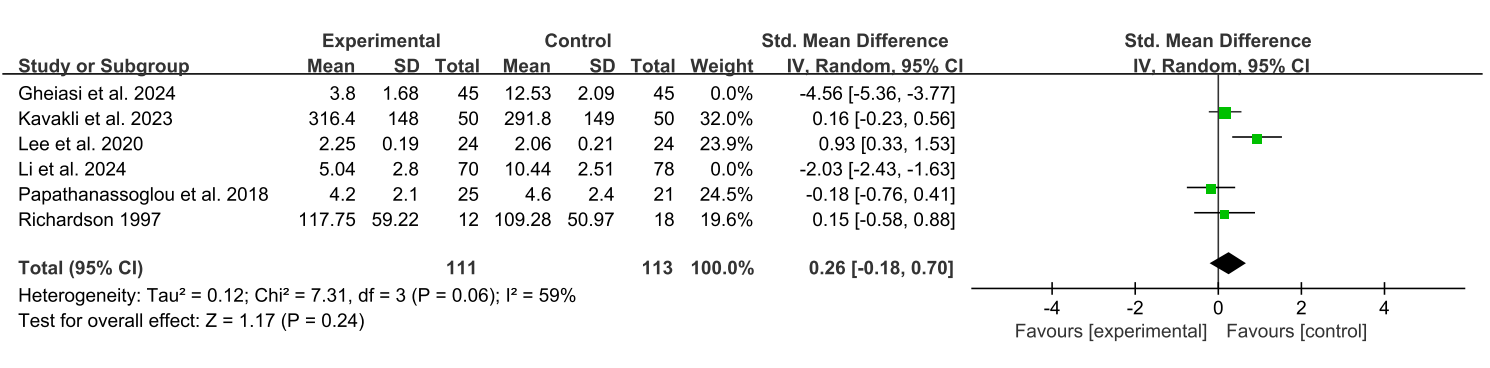
Supplementary 24

Forest plot of the meta-analysis on patients' sleep quality (post-intervention) –sensitivity analysis


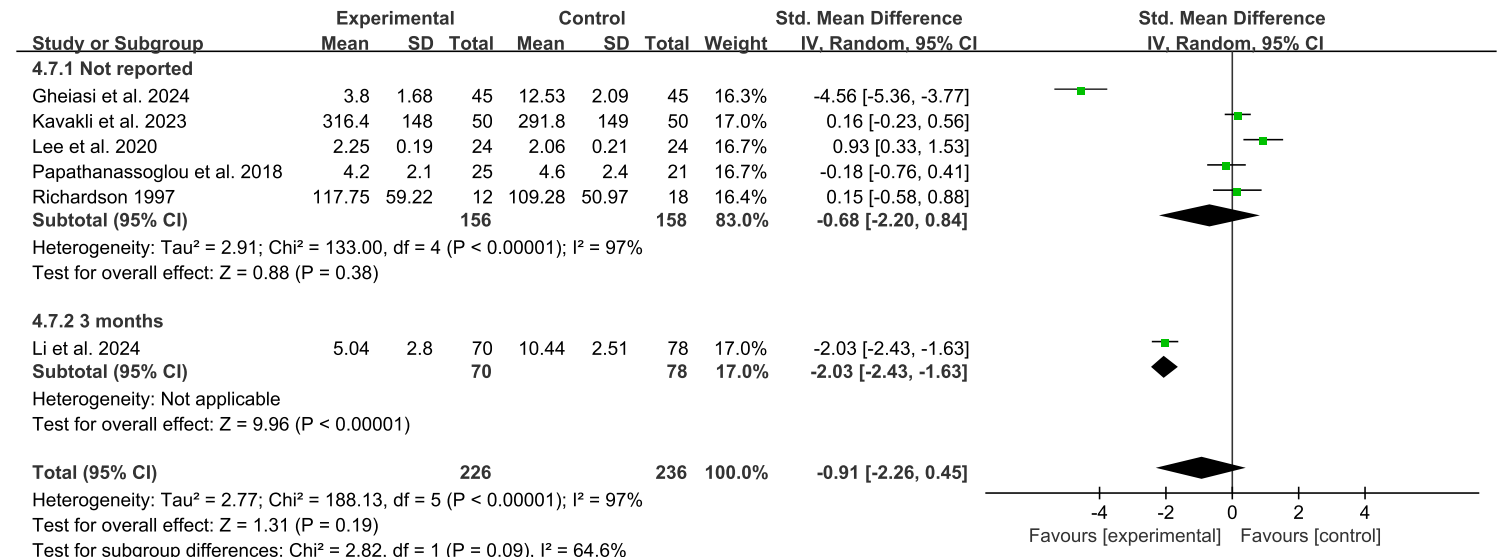
 Supplementary 25

Forest plot of the meta-analysis on patients' sleep quality (post-intervention) –subgroup by follow-up duration


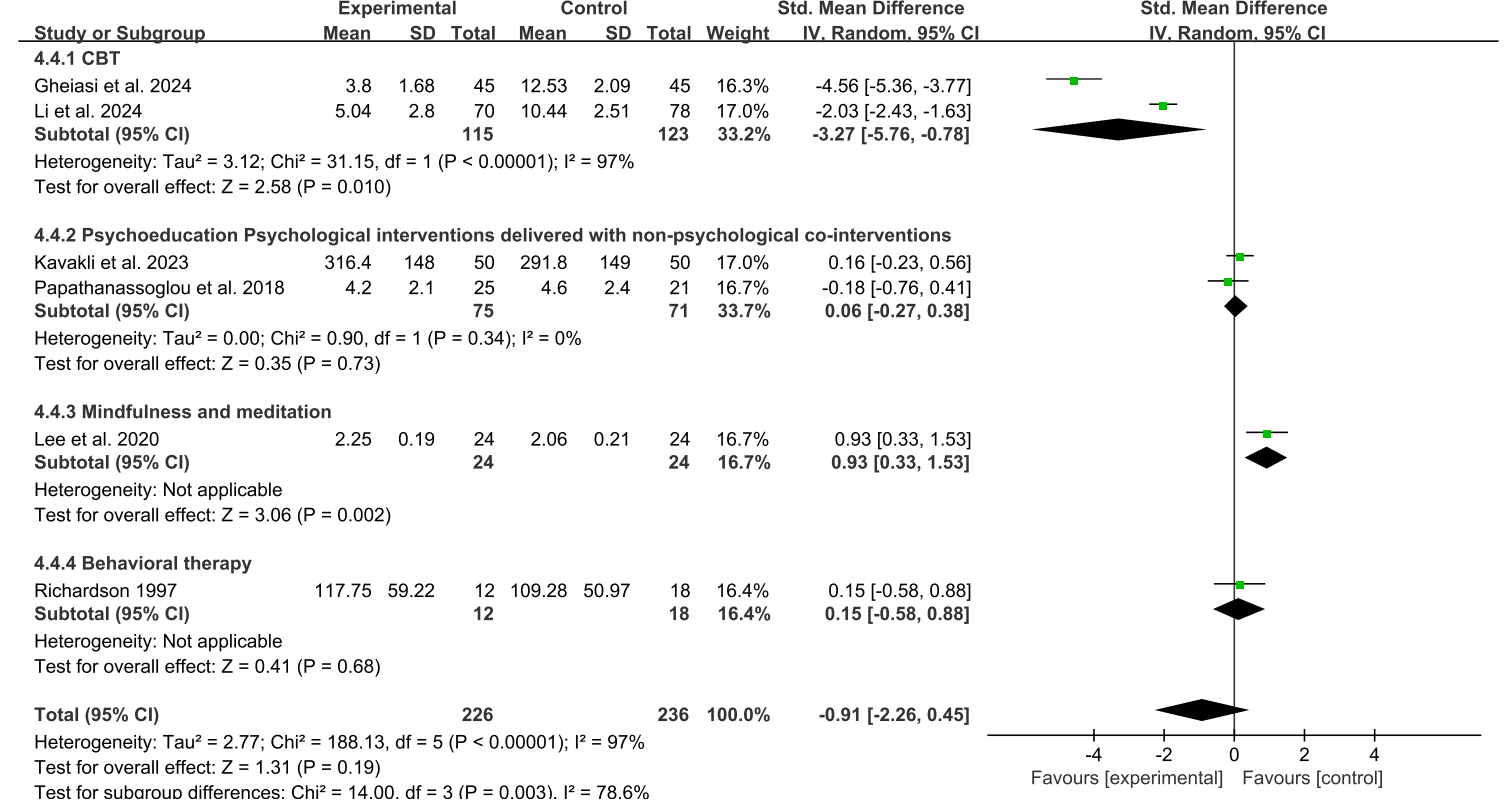
 Supplementary 26

Forest plot of the meta-analysis on patients' sleep quality (post-intervention) –subgroup by type of psychological intervention


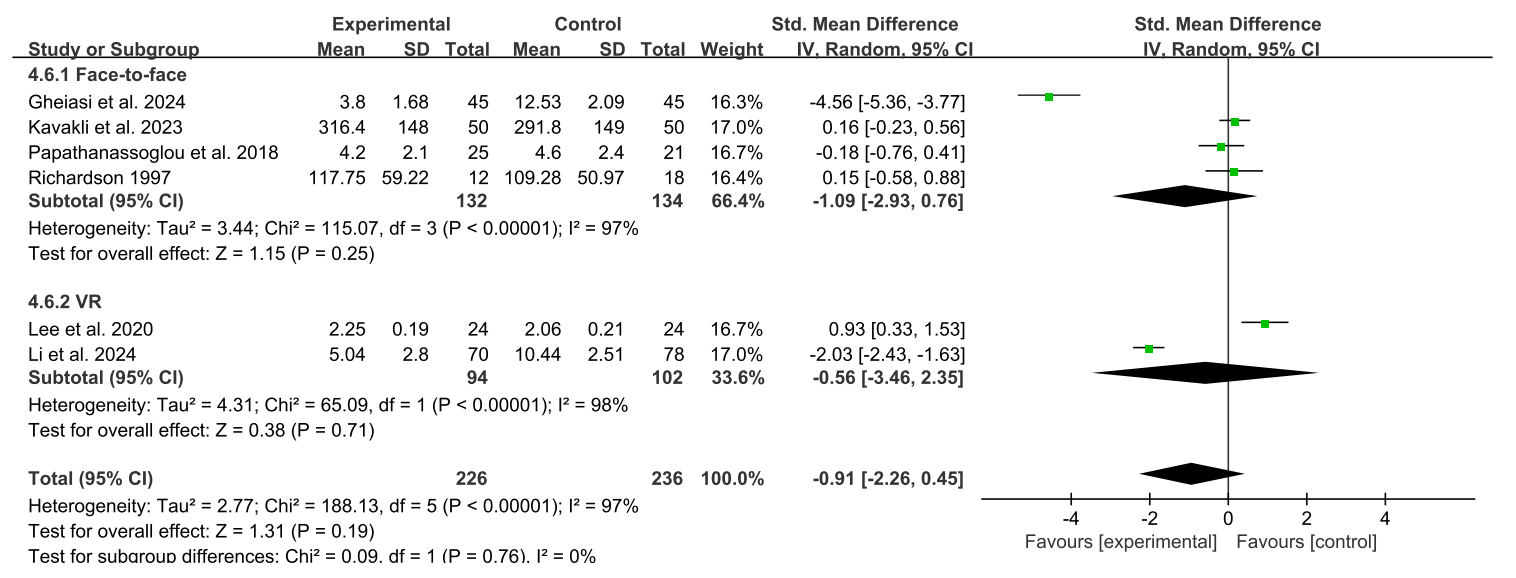
 Supplementary 27

Forest plot of the meta-analysis on patients' sleep quality (post-intervention) –subgroup by mode of delivery


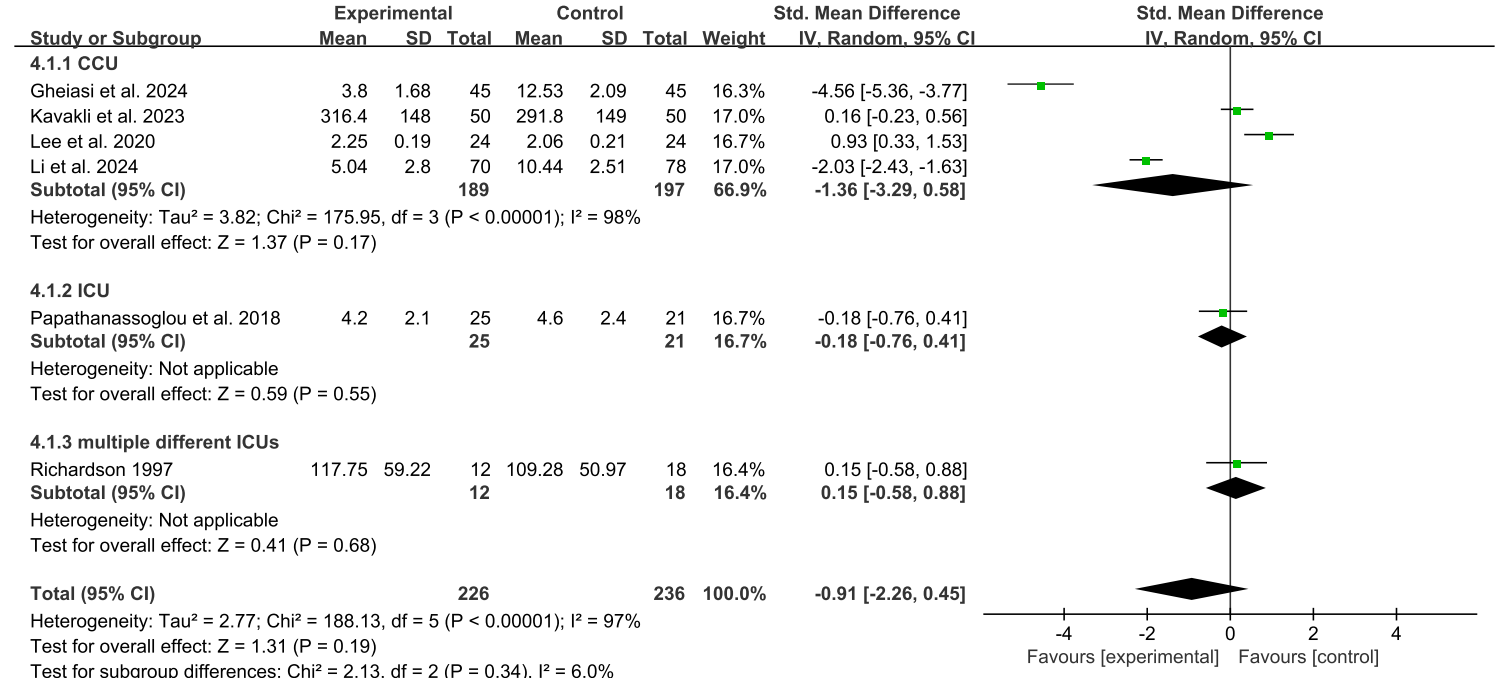
 Supplementary 28

Forest plot of the meta-analysis on patients' sleep quality (post-intervention) –subgroup by ICU setting


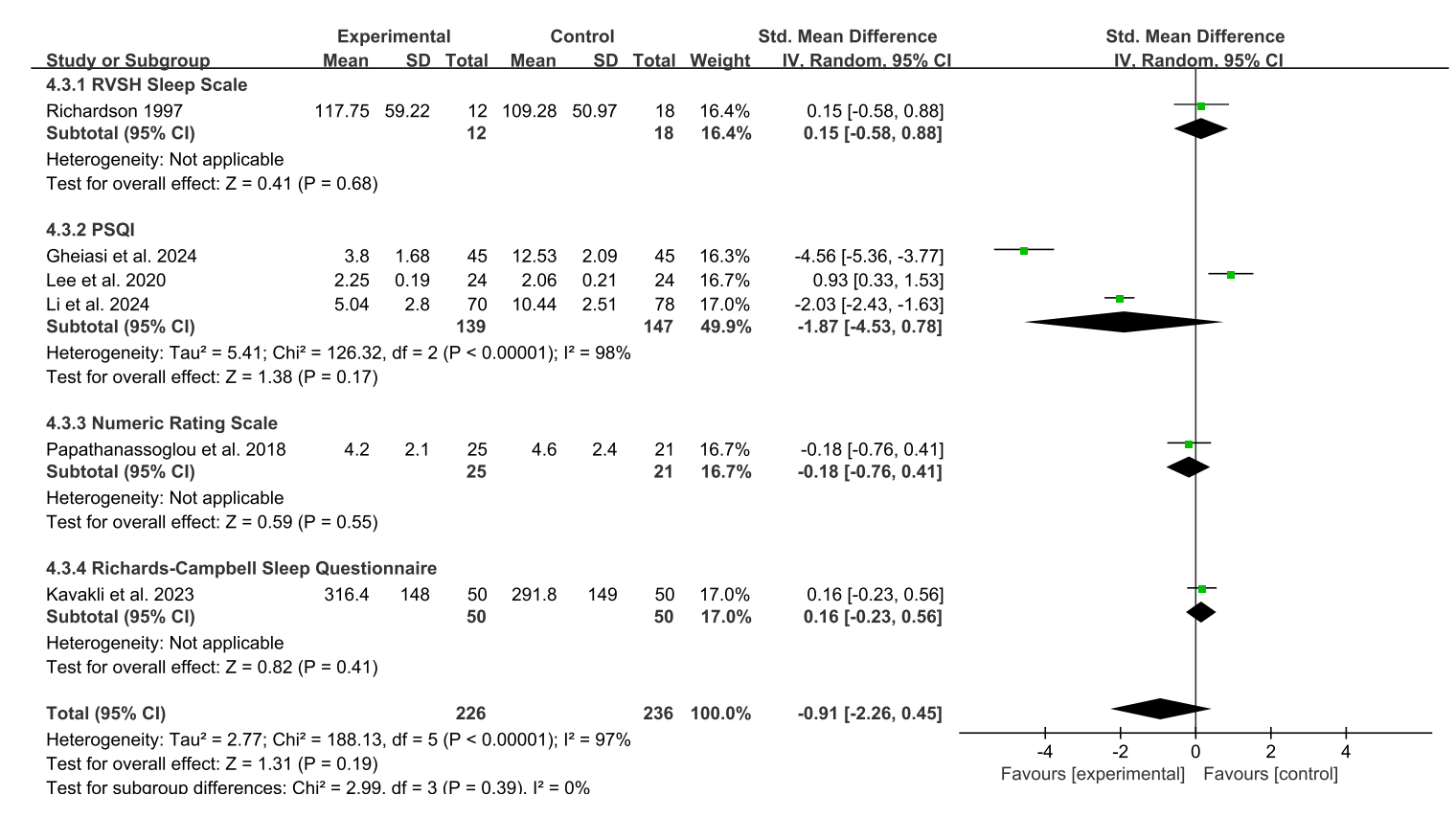
 Supplementary 29

Forest plot of the meta-analysis on patients' sleep quality (post-intervention) –subgroup by assessment tool


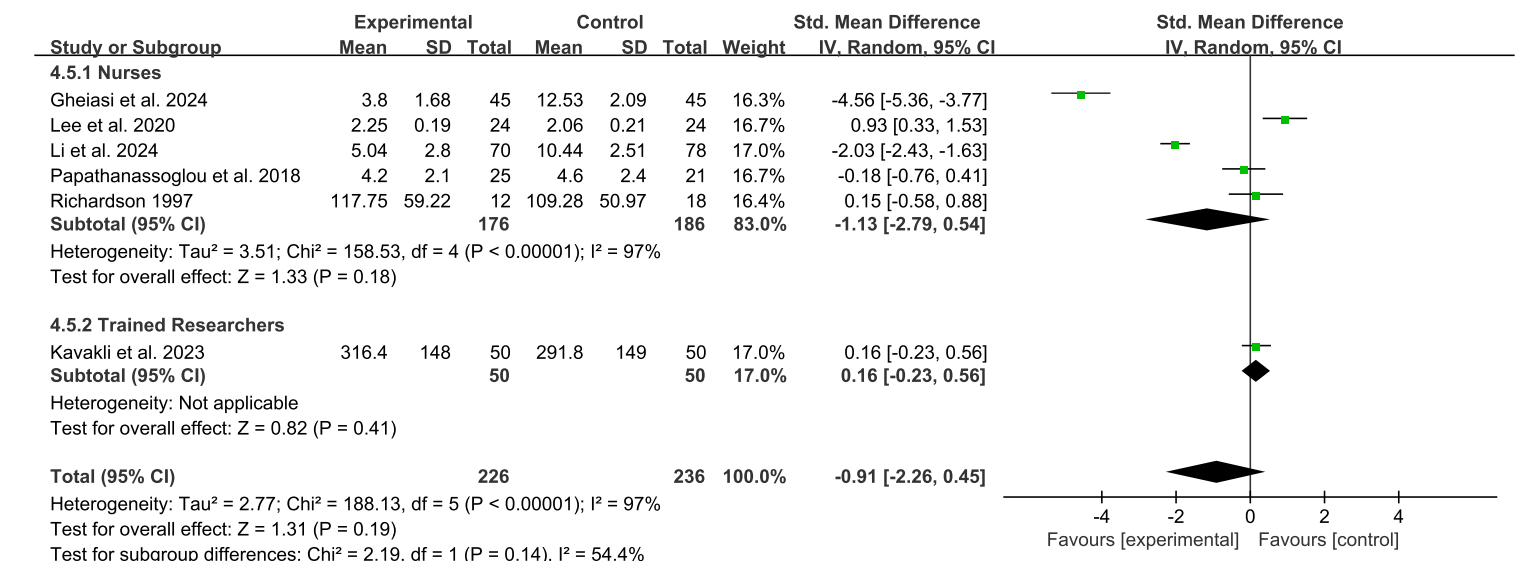


Supplementary 30

Forest plot of the meta-analysis on patients' sleep quality (post-intervention) –subgroup by provider expertise


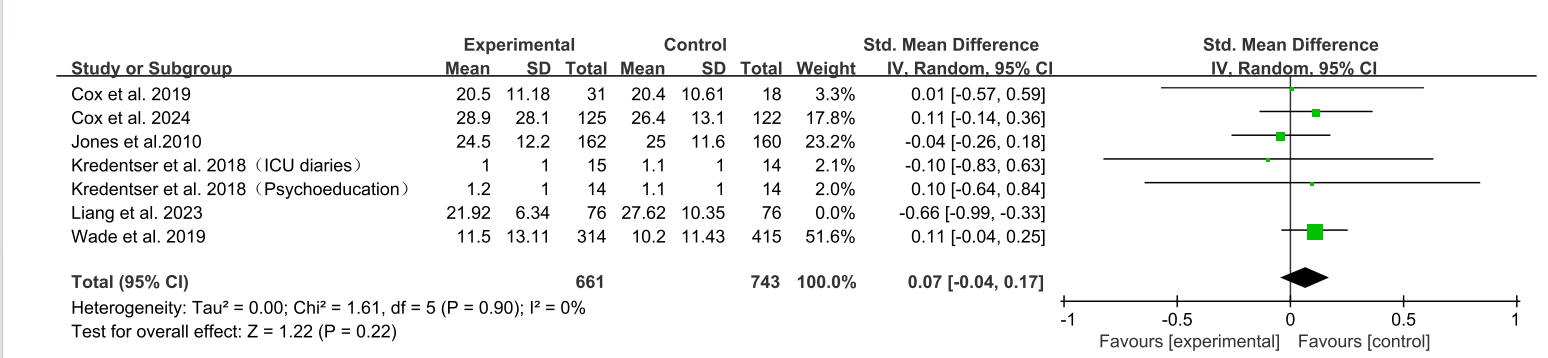
Supplementary 31

Forest plot of the meta-analysis on patients' PTSD (post-intervention) –sensitivity analysis


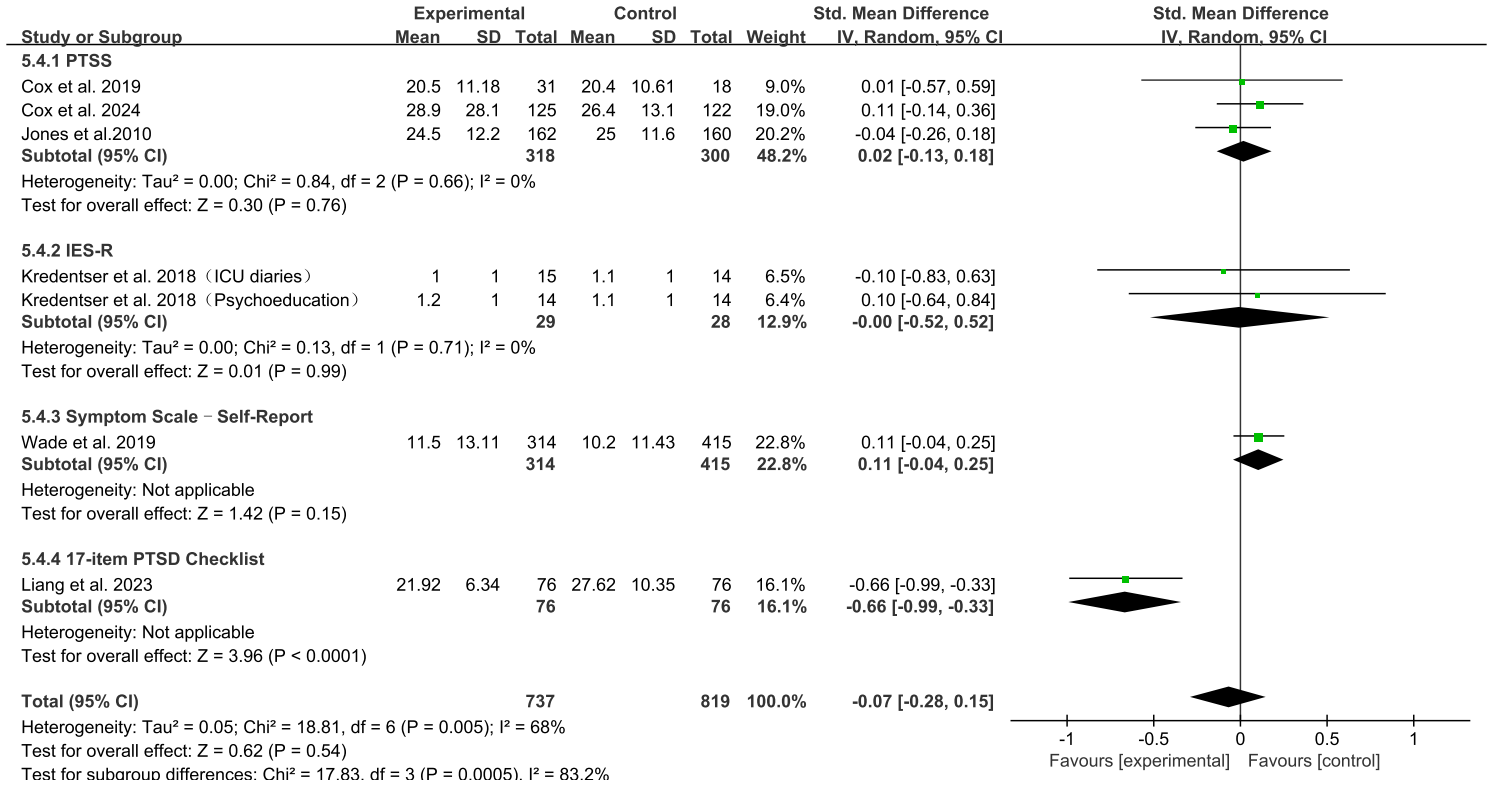
 Supplementary 32

Forest plot of the meta-analysis on patients' PTSD (post-intervention) –subgroup by assessment tool


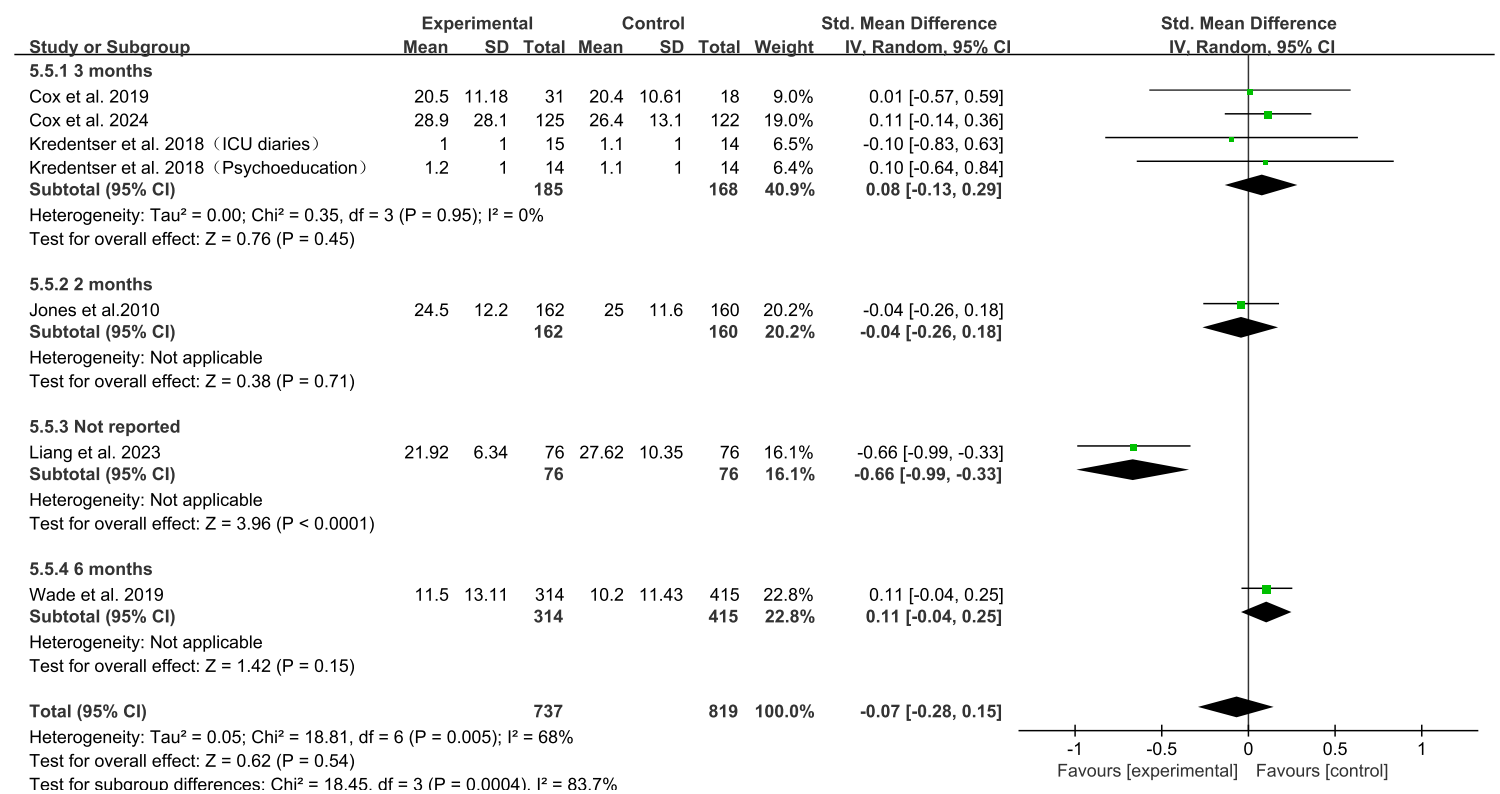
 Supplementary 33

Forest plot of the meta-analysis on patients' PTSD (post-intervention) –subgroup by follow-up duration


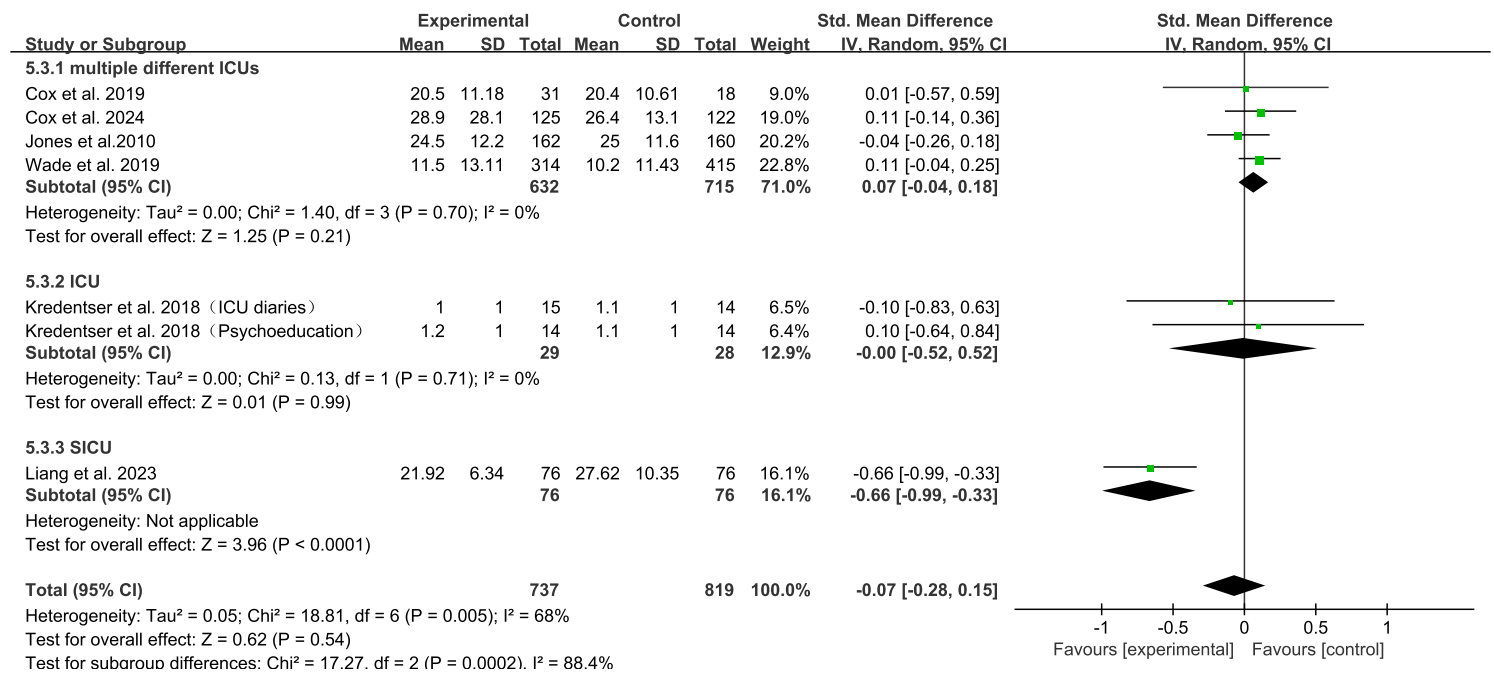
 Supplementary 34

Forest plot of the meta-analysis on patients' PTSD (post-intervention) –subgroup by ICU setting


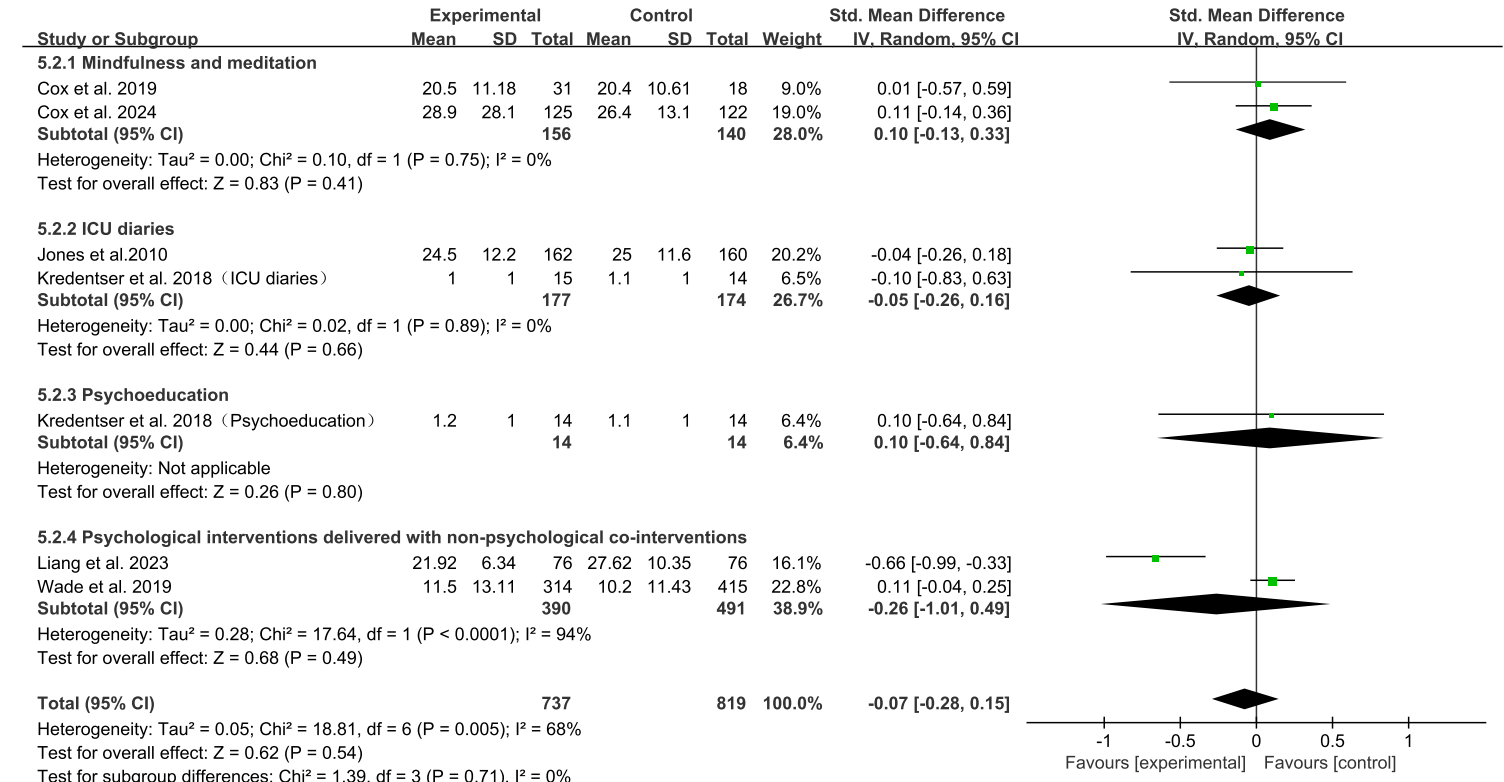
 Supplementary 35

Forest plot of the meta-analysis on patients' PTSD (post-intervention) –subgroup by ICU setting


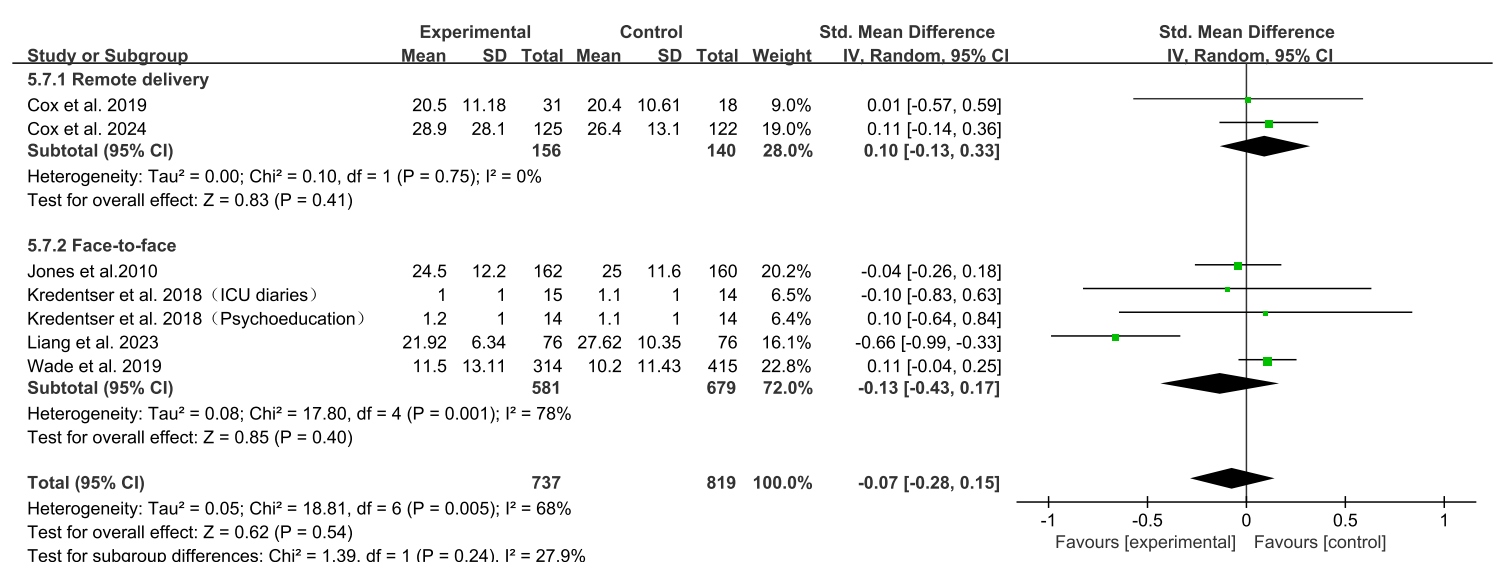
 Supplementary 36

Forest plot of the meta-analysis on patients' PTSD (post-intervention) –subgroup by mode of delivery


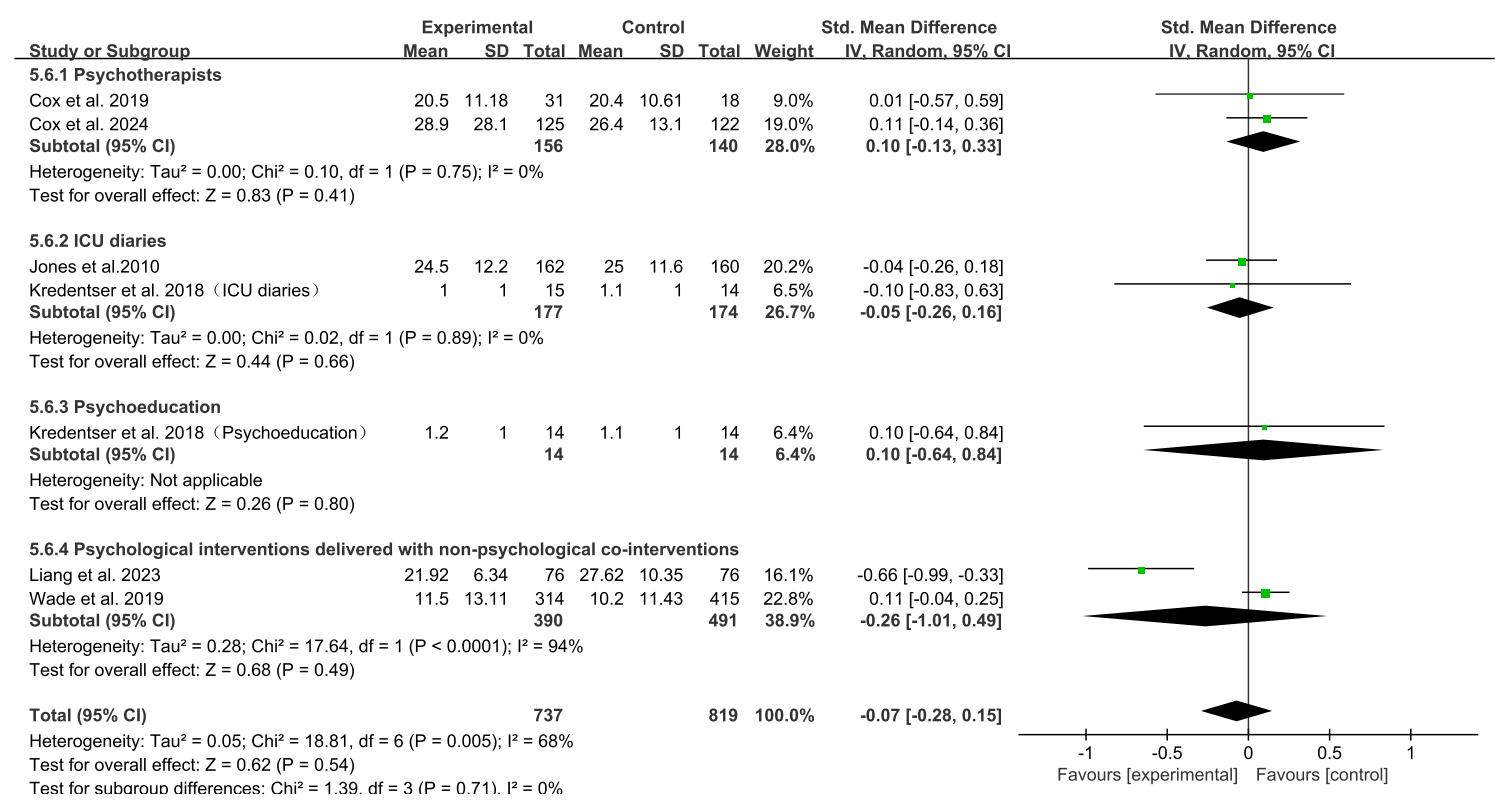
 Supplementary 37

Forest plot of the meta-analysis on patients' PTSD (post-intervention) –subgroup by provider expertise


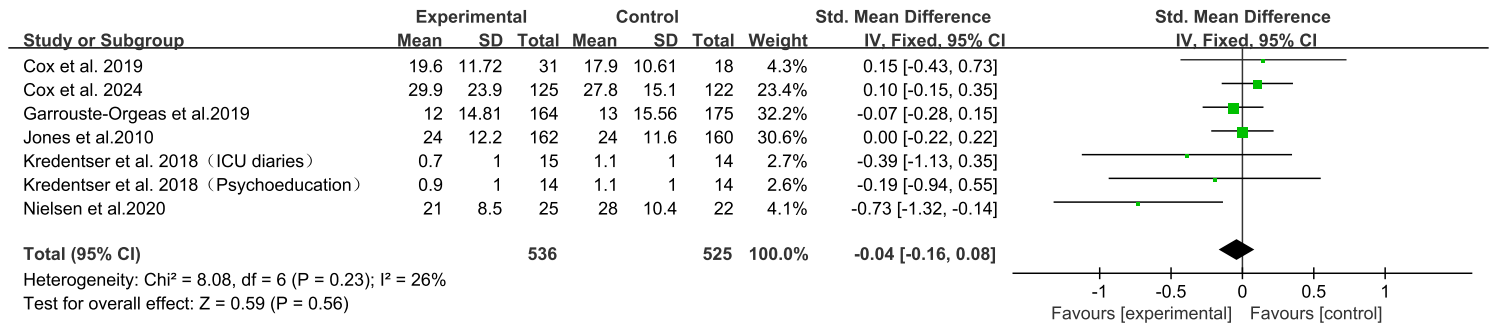
Supplementary 38

Forest plot of the meta-analysis on patients' PTSD (short-term follow-up)


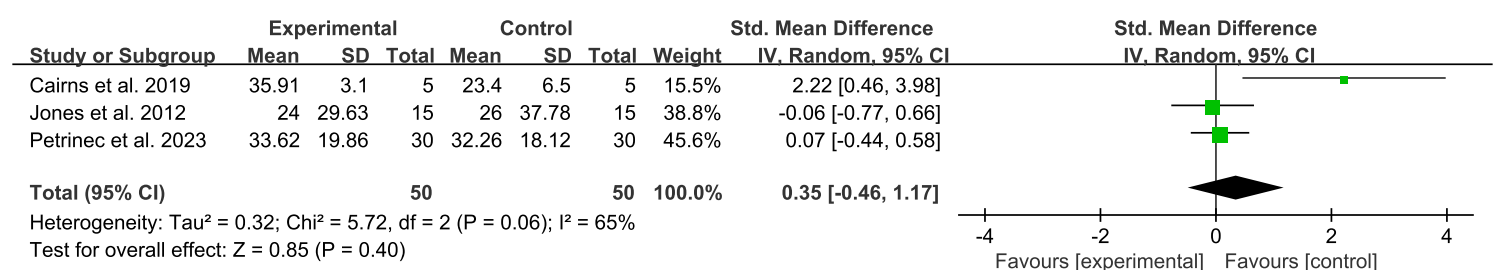
Supplementary 39

Forest plot of the meta-analysis on PTSD in family members (post-intervention)


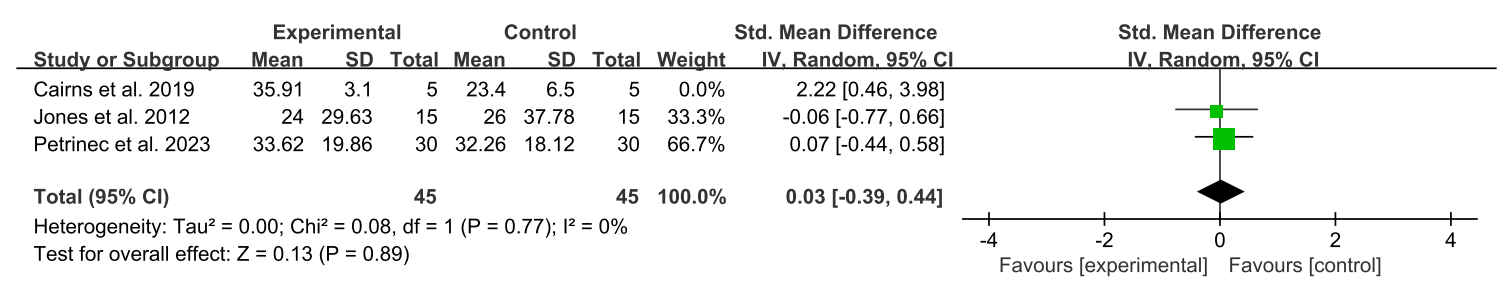
 Supplementary 40

Forest plot of the meta-analysis on PTSD in family members (post-intervention)- sensitivity analysis


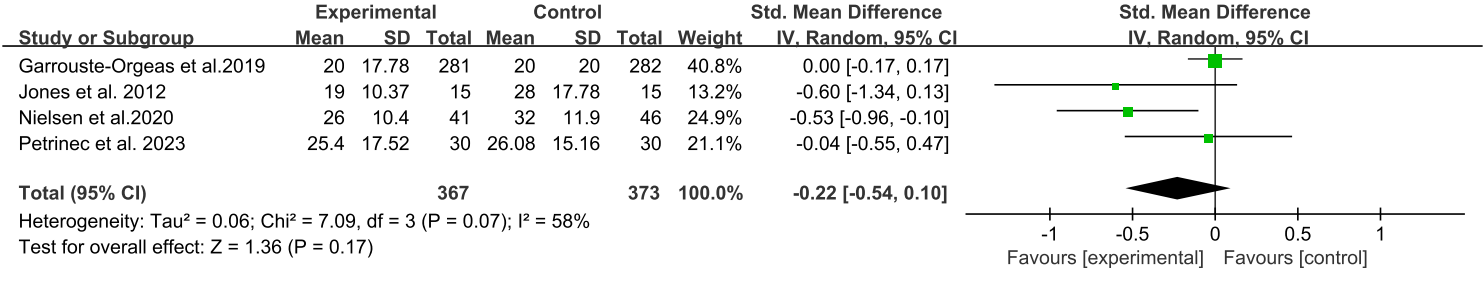


Supplementary 41

Forest plot of the meta-analysis on PTSD in family members (short-term follow-up)


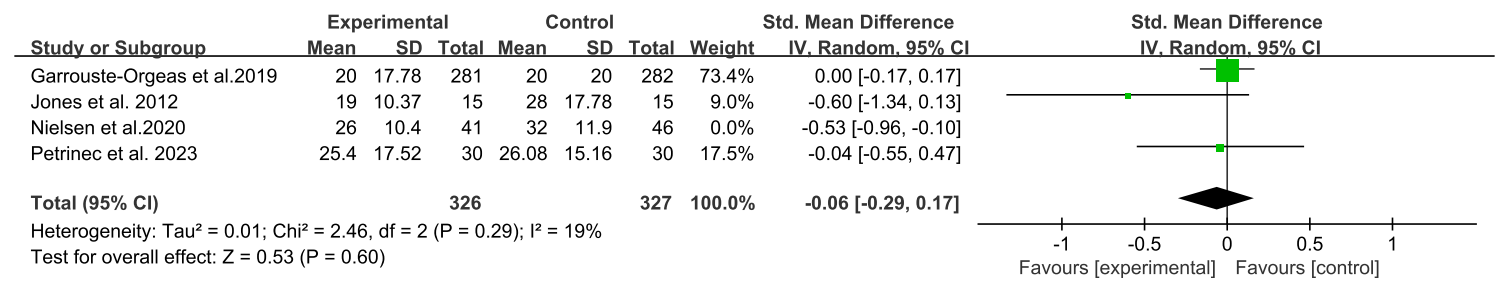
Supplementary 42

Forest plot of the meta-analysis on PTSD in family members (short-term follow-up)- sensitivity analysis


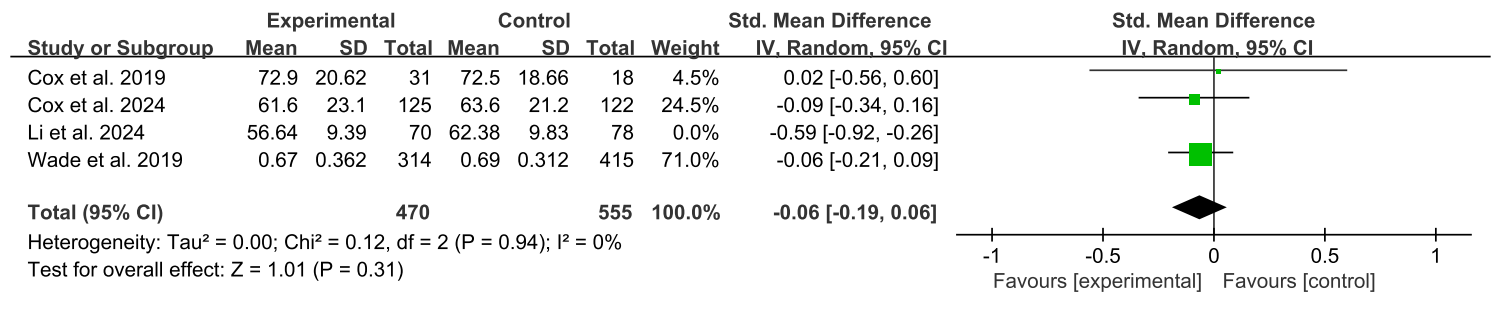


Supplementary 43

Forest plot of the meta-analysis on patients' quality of life (post-intervention) - sensitivity analysis


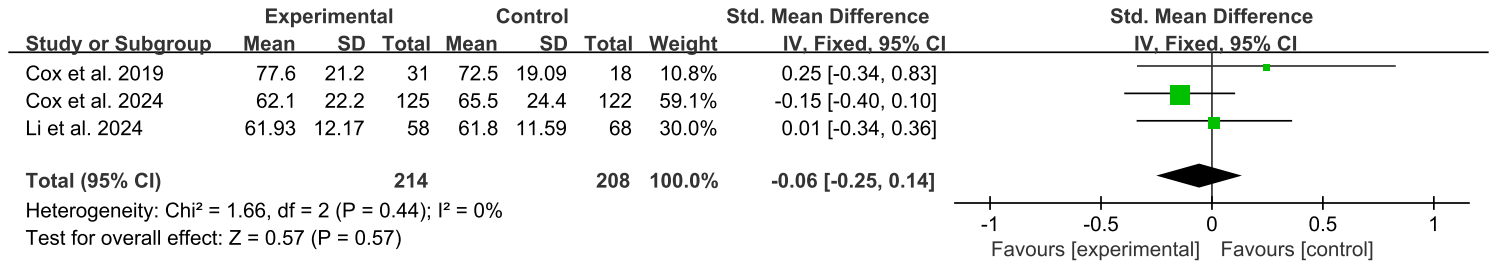
Supplementary 44

Forest plot of the meta-analysis on patients' quality of life (short-term follow-up)


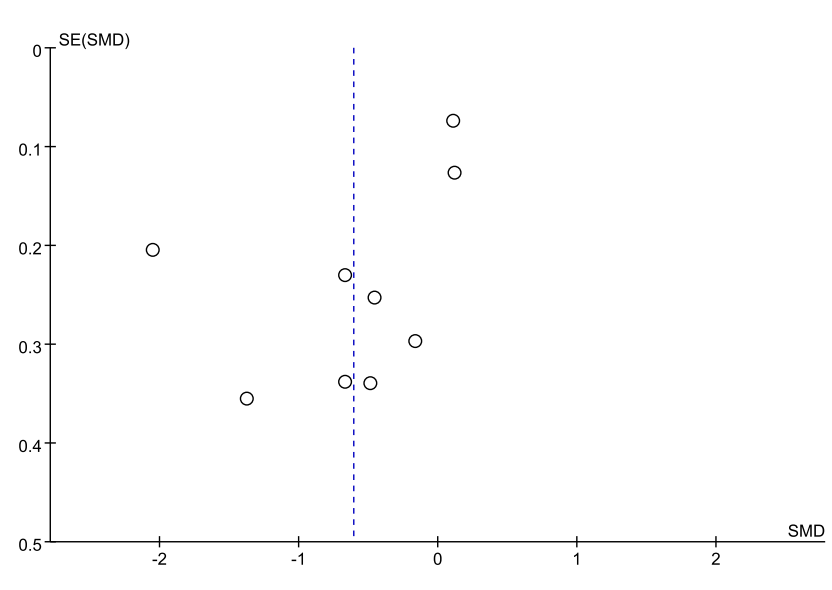
Supplementary 45

The funnel plot of the immediate effect of anxiety


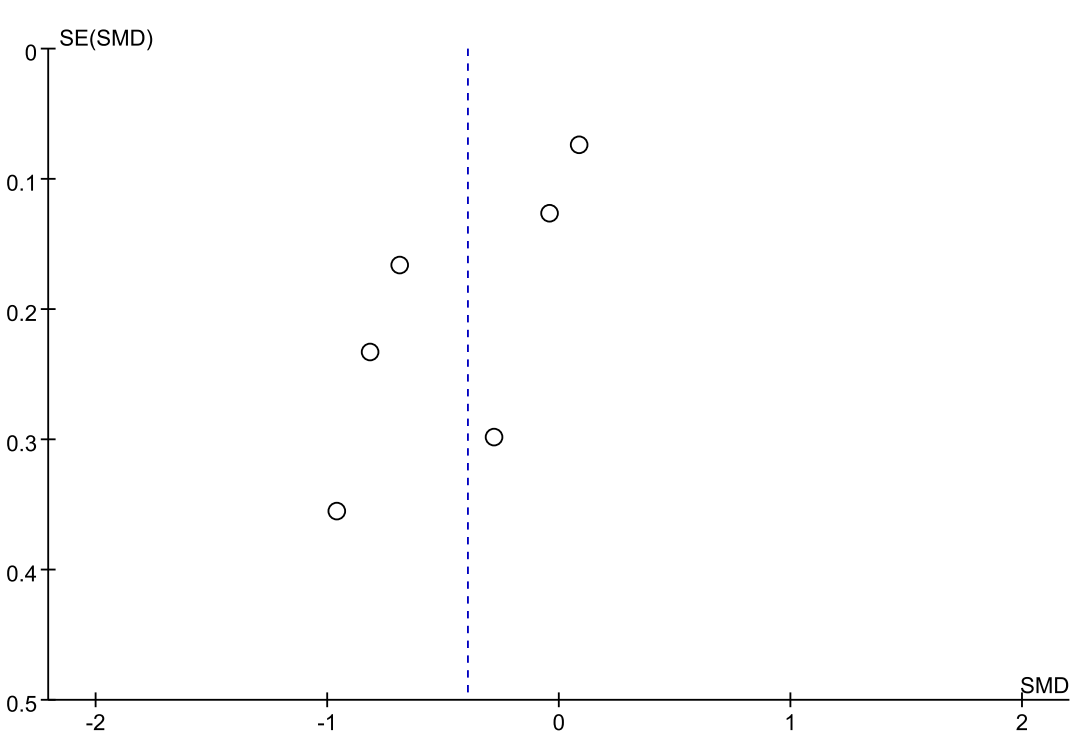
Supplementary 46

The funnel plot of the immediate effect of depression


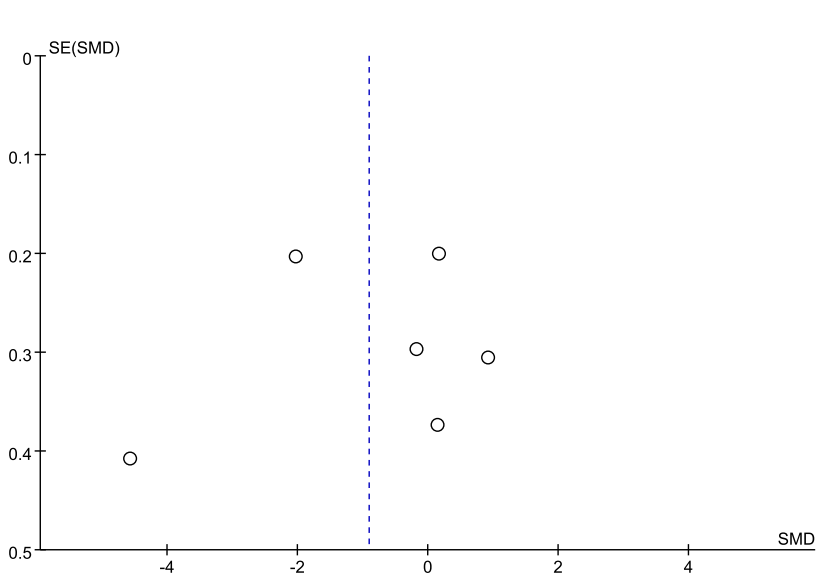
 Supplementary 47

The funnel plot of the immediate effect of sleep quality


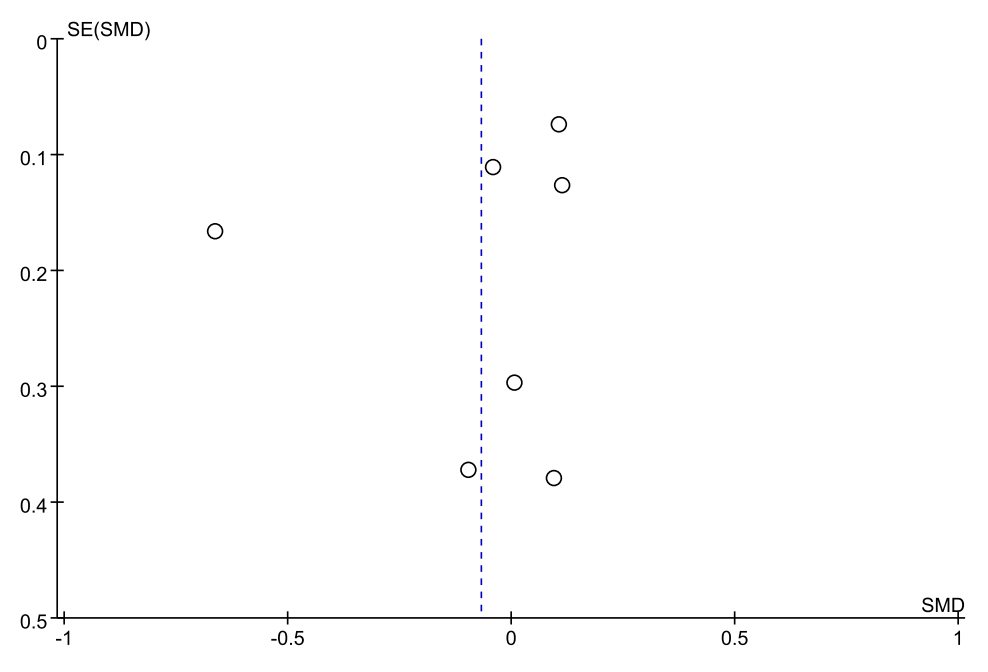
 Supplementary 48

The funnel plot of the immediate effect of PTSD


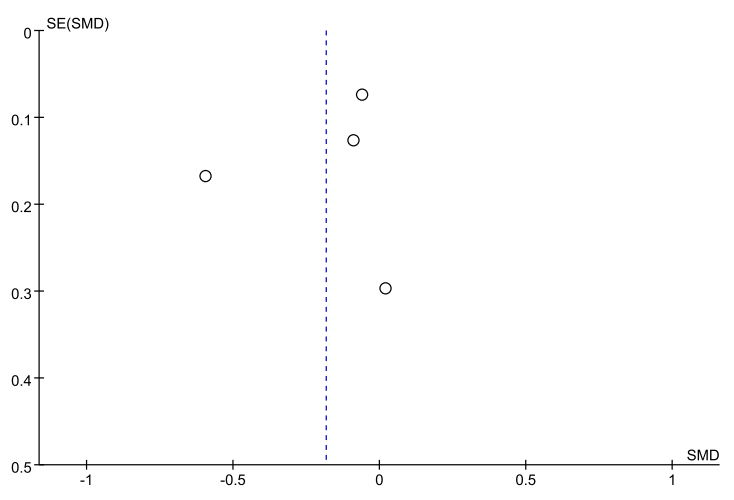


Supplementary 49

The funnel plot of the immediate effect of quality of life
